# Genome-wide identification of the BASS gene family in four *Gossypium* species and functional characterization of *GhBASSs* against salt stress

**DOI:** 10.1038/s41598-021-90740-3

**Published:** 2021-05-31

**Authors:** Thwin Myo, Fang Wei, Honghao Zhang, Jianfeng Hao, Bin Zhang, Zhixian Liu, Gangqiang Cao, Baoming Tian, Gongyao Shi

**Affiliations:** 1 Research Base, State Key Laboratory of Cotton Biology, Zhengzhou University, Zhengzhou, 450001 Henan China; 2 Henan International Joint Laboratory of Crop Gene Resources and Improvement, School of Agricultural Sciences, Zhengzhou University, Zhengzhou, 450001 Henan China

**Keywords:** Biotechnology, Computational biology and bioinformatics, Molecular biology, Plant sciences

## Abstract

Bile acid sodium symporter (BASS) family proteins encode a class of sodium/solute symporters. Even though the sodium transporting property of BASSs in mammals was well studied, their sodium transportability and functional roles in plant salt tolerance remained largely unknown. Here, BASS family members from 4 cotton species, as well as 30 other species were identified. Then, they were designated as members of BASS1 to BASS5 subfamilies according to their sequence similarity and phylogenetic relationships. There were 8, 11, 16 and 18 putative BASS genes in four cotton species. While whole-genome duplications (WGD) and segmental duplications rendered the expansion of the BASS gene family in cotton, BASS gene losses occurred in the tetraploid cotton during the evolution from diploids to allotetraploids. Concerning functional characterizations, the transcript profiling of *GhBASSs* revealed that they not only preferred tissue-specific expression but also were differently induced by various stressors and phytohormones. Gene silencing and overexpression experiments showed that *GhBASS1* and *GhBASS3* positively regulated, whereas *GhBASS2*, *GhBASS4* and *GhBASS5* negatively regulated plant salt tolerance. Taken together, BASS family genes have evolved before the divergence from the common ancestor of prokaryotes and eukaryotes, and *GhBASSs* are plastidial sodium-dependent metabolite co-transporters that can influence plant salt tolerance.

## Introduction

Salinity stress is a global environmental challenge and one of the major constraints decreasing crop production worldwide^1,2^. Munns^3^ reported that twenty percent of agronomically utilized and irrigated land suffers from sodium (Na^+^) contamination, which imposes osmotic stress and ion toxicity on plants. Furthermore, the high level of Na^+^ inside the cells provokes imbalances of K^+^/Na^+^ homeostasis, further disturbance of physiological processes and also the confusion of salt-responsive genes expression, leading to cell damage, growth arrest and even the death of plant cells^4^. Since Na^+^ is the main culprit behind salt stress, it is demanded to gain insights into the identities of genes encoding Na^+^ transporters and channels that pertain to Na^+^. Several salt-tolerant genes regulating K^+^/Na^+^ homeostasis have been identified in recent years, e.g., *Arabidopsis* K^+^ transporter (AKT1), high affinity K^+^ transporters (HKTs), Na^+^/H^+^ exchangers (NHXs) and salt overly sensitive (SOS1). Therefore, further insights into the identities and properties of sodium specific transporters, especially those whose functions are Na^+^-specifically dependent, are vital for us to understand salt stress and tolerant mechanisms in plants.

Bile acid sodium symporters (BASS) belong to the solute carrier family 10 (SLC10) that were first identified as the Na^+^-taurocholate co-transporter, NTCP, (SLC10A1) in the mammalian liver^5^. In humans, bile acids (BA) are synthesized in the liver and stored in the gallbladder. In response to meal ingestion, BA are secreted from the gallbladder to the intestine as detergents to aid the solubilization and digestion of dietary nutrients, including vitamins and cholesterol^6^. Then, BA are reabsorbed by the apical sodium-dependent bile acid transporter, ASBT, (SLC10A2) in the small intestine. And through the portal circulation, BA are reloaded in the liver by the sodium-dependent NTCP^6^. Both ASBT and NTCP carry out the uphill transport of bile acids across membranes with the use of Na^+^ gradient^7^. The crystal structures of ASBT_NM_ and ASBT_Yf_ (ASBT homologues from *Neisseria meningitidis* and *Yersinia frederiksenii*) provided a structural basis for the Na^+^-dependent symporting mechanism of BASS. BASS is composed of ten transmembrane segments (TM1-10) and two conserved Na^+^-binding sites, Na1 and Na2^8^. Bonding of two sodium ions specifically to Na^+^-binding sites is a prerequisite for the structural changes and substrate-binding of BASS^8^.

Further analyses discovered that BASS family members are widely distributed across all organisms, including bacteria, fungi, plants and animals. Even though the residues forming the two Na^+^-binding sites are highly conserved, BASSs show a broad range of substrate specificity. For example, NTCP (SLC10A1) and ASBT (SLC10A2) are primarily bile acid transporters, and the sodium-dependent organic anion transporter (SOAT, SLC10A6) primarily transports sulfated steroids. Moreover, NTCP was also shown to transport steroids and xenobiotics^6^. Intriguingly, although bile acids are not produced in plants, BASS family genes are present in all genomes of plants that have been characterized so far^9^. For instance, *Arabidopsis* contains six BASS members, namely *AtBASS1* to *AtBASS6*. *AtBASS1* was annotated as a bile acid symporter involved in the transport of pantoate. *AtBASS2* was reported to serve as a sodium-coupled pyruvate transporter in plastid envelopes^9^. In addition, *AtBASS5* was a plastidial transporter of 2-keto-4 methylthiobutyrate (KMTB) which was needed for the synthesis of methionine-derived glucosinolates in *Arabidopsis*^10,11^. South et al.^12^ stated that *AtBASS6* was also localized in the chloroplast envelope and transported glycolates during photorespiration in *Arabidopsis*. Therefore, all *Arabidopsis* BASSs might play as sodium-coupled metabolite transporters in plastid envelopes, even though the substrate specificity of some BASS members remains undetermined.

The conserved maintenance of BASSs in plants indicates the widespread importance of Na^+^-coupled metabolite transport in plant plastids and potential roles of BASSs in plant Na^+^ physiology. In spite of the fact that the sodium transporting mechanism of BASSs in mammals was well studied, there was little information about the effect of BASSs on Na^+^ homeostasis in plants. To the best of our knowledge, only one study stated that *TaBASS2* positively regulated salinity tolerance in wheat via modulation of ABI4 expression^13^. Recently, the BASS gene family has been identified in some plants such as *Arabidopsis thaliana*, *Flaveria bidentis*, *Oryza stiva* and *Sorghum bicolor*^9^. However, there was a lack of genome-wide identification and functional characterizations of the BASS gene family in cotton, especially a systematical investigation of their roles in salt stress responses.

Cotton (*Gossypium* spp.) is one of the most important fibers and oil crops, as well as it is grown in more than 80 countries, providing jobs for about 100 million family units with the economic impact of approximately US $500 billion per year worldwide^14^. The *Gossypium* genus is composed of 50 species in which four cultivated cotton species such as *G. hirsutum* L. (AD)1 and *G. barbadense* L. (AD)2, *G. herbaceum* L. (A1) and *G. arboreum* L. (A2) have been domesticated independently^15^. Among them, the allotetraploid Upland cotton (*G. hirsutum*) is the most significant and accounts for 95% of worldwide cotton production^14^. As we know, the yield and quality of cotton are severely threatened by salinity stress^16^. Hence, salt-induced decrease in yield and quality of cotton alarms cotton researchers to mine strategies for enhancing salt tolerance of cotton. Such a situation inspires us to identify the cotton BASS gene family and elucidate functions of *G. hirsutum* BASS genes at the molecular level.

The completion of genome sequencing for four *Gossypium* species provides a valuable resource for genome-wide identification and computational analyses of gene families in cotton^17–20^. Hence, it will be of much valuable significance to study BASS family genes in cotton and evaluate their functional roles in cotton salt tolerance. Here, we identified 8 putative BASSs from *G. arboreum*, 11 from *G. raimondii*, 16 from *G. hirsutum* and 18 from *G. barbadense*, respectively. Cotton BASS gene structures, phylogenetic relationships, evolutionary history and other related analyses were systematically conducted by employing bioinformatics technologies. Moreover, the transcript profiles of *GhBASSs* were studied not only in different tissues but also in response to abiotic stresses and hormone elicitors. Furthermore, we examined the effects of *GhBASSs* on plant Na^+^ homeostasis and salt stress tolerance through gene silencing and gene overexpression. Our results demonstrated that BASS family genes were very conserved across prokaryotes as well as eukaryotes, and *GhBASSs* were certainly involved in the cotton response to salt stress, but their regulation and response mechanisms are different with respect to salt stress.

## Results

### Identification and phylogenetic analysis of BASS family members in different species

In this study, our main focus was four cotton species, so we first compared publicly available genome databases and finally selected Beijing Genomics Institute sequenced genome (BGI) for *G. arboretum*, Plant Genome Mapping laboratory (University of Georgia) sequenced genome (JGI) for *G. raimondii*, Nanjing Agricultural University sequenced genome (NAU-NBI) for *G. hirsutum* and Chinese National Human Genome Center sequenced genome (CHGC) for *G. barbadense*. After eliminating redundant sequences, 7 putative BASS genes and 2 pseudogenes from the *G. arboretum* genome, 11 putative BASS genes and 1 pseudogene from the *G. raimondii* genome, 15 putative BASS genes from the *G. hirsutum* genome and 18 putative BASS genes and 3 pseudogenes from the *G. barbadense* genome were respectively identified by performing the hmmsearch to their respective protein sequence databases. Since the released genome and annotation were not flawless, these BASS gene sequences were revised by comparing them with the genomic sequences, followed by correction and integration with the data from EST and SRA databases. After extensive bioinformatics analyses, a total of 8 and 11 putative BASS genes from the genomes of diploid species (*G. arboretum* and *G. raimondii*) and 16 and 18 putative BASS genes from the genomes of tetraploid species (*G. hirsutum* and *G. barbadense*) were retrieved (Tables 1, S1, S2). After performing Pfam (http://pfam.xfam.org/) and NCBI-CDD (https://www.ncbi.nlm.nih.gov/Structure/cdd/ wrpsb.cgi), all 53 candidate genes retained the conserved SBF and bass domain (Fig. S1), which validates the typical configuration of BASS family members. What is more, transmembrane regions and Na^+^-binding sites are unique characteristics of BASS genes, and all these candidates possessed these qualities also (Fig. S2).

**Table 1:** Characteristics of putative BASS genes from four cotton species.

	*G. arboreum/G. raimondii*				*G. barbadense*				*G. hirsutum*			
	Gene Name	Gene ID	Coordinate	AA	Gene Name	Gene ID	Coordinate	AA	Gene Name	Gene ID	Coordinate	AA
BASS1	GaBASS1-1	Cotton_A_25338	AA07: 93,472,010…93,474,430 +	403	GbBASS1-1A	GOBAR_AA27322	At01: 4,600,283…4,602,708 −	403	GhBASS1-1A	Gh_A01G0308	At01: 4,029,556…4,035,035 −	314
					gbbass1-1A*	*GOBAR_AA27321*	*At01: 4,597,765…4,598,584* −	*173*				
	GrBASS1-1	Gorai.002G045800.1	DD02: 3,816,743…3,819,556 −	403	GbBASS1-1D	GOBAR_DD11480	Dt01: 3,655,786…3,658,211 +	411	GhBASS1-1D	Gh_D01G0342	Dt01: 3,713,612…3,716,034 −	400
	GaBASS1-2	Cotton_A_26041	AA12: 121,943,193…121,944,861 +	260	GbBASS1-2A	GOBAR_AA06322	At04: 58,979,100…58,984,268 −	528	GhBASS1-2A	Gh_A04G1146	At04: 61,734,341…61,739,690 −	405
	GrBASS1-2	Gorai.012G169500.1	DD12: 33,976,519…33,978,953 −	405	GbBASS1-2D	GOBAR_DD37643	Dt04: 47,647,909…47,649,054 −	244	GhBASS1-2D	Gh_D04G1759	Dt04: 49,841,451…49,849,537 −	379
	grbass1-2*	*Gorai.012G168600.1*	*DD12: 33,881,190…33,882,278* −	*103*	gbbass1-2D*	*GOBAR_DD33853*	*Dt04: 47,973,990…47,975,424* −	*193*				
BASS2	GaBASS2-1	Cotton_A_20435	AA08: 78,899,369…78,903,181 −	417	GbBASS2-1A	GOBAR_AA00062	At06: 12,834,018…12,839,074 +	417	GhBASS2-1A	Gh_A06G0507	At06: 10,259,748…10,263,572 +	443
	GrBASS2-1	Gorai.010G067700.1	DD10: 8,783,351…8,787,641 +	417	GbBASS2-1D	GOBAR_DD09546	Dt06: 9,649,301…9,653,128 +	417	GhBASS2-1D	Gh_D06G0564	Dt06: 8,853,217…8,857,042 +	418
	GaBASS2-2	Cotton_A_03100	AA13: 82,738,501…82,743,178 +	416	GbBASS2-2A	GOBAR_AA05834	At13: 104,039,478…104,039,480 −	416	GhBASS2-2A	Gh_A13G1752	At13: 76,390,663…76,395,343 −	416
	GrBASS2-2	Gorai.013G231400.1	DD13: 54,983,262…54,988,024 −	443	GbBASS2-2D	GOBAR_DD22451	Dt13: 56,239,584…56,244,283 +	424	GhBASS2-2D	Gh_D13G2100	Dt13: 56,468,734…56,473,336 −	373
BASS3	GaBASS3-1	Cotton_A_15215	AA01: 128,242,749…128,245,983 +	434	GbBASS3-1A	GOBAR_AA01976	At07: 87,375,132…87,378,356 +	434	GhBASS3-1A	Gh_A07G1785	At07: 72,610,458…72,613,682 +	434
	GrBASS3-1	Gorai.001G228400.1	DD01: 46,480,755…46,484,530 +	425	GbBASS3-1D	GOBAR_DD22389	Dt07: 50,073,715…50,077,872 +	469	GhBASS3-1D	Gh_D07G1990	Dt07: 49,517,367…49,520,454 +	425
	GaBASS3-2	Cotton_A_03247	AA03: 27,356,636…27,359,507 −	415	GbBASS3-2A	GOBAR_AA26078	At08: 6,554,204…6,559,561 −	254	GhBASS3-2A	\	At08: 5,886,270…5,889,135 −	348
	gabass3-2a*	*Cotton_A_03245*	*AA03: 27,324,813…27,329,152* −	*179*	gbbass3-2A*	*GOBAR_AA26076*	*At08: 6,542,938…6,543,589* −	*146*				
	gabass3-2b*	*Cotton_A_03243*	*AA03: 27,313,725…27,314,180* −	*112*								
	GrBASS3-2a	Gorai.004G059600.1	DD04: 5,786,233…5,789,721 −	415	GbBASS3-2 Da	GOBAR_DD02252	Dt08: 6,151,808…6,154,671 −	412	GhBASS3-2D	Gh_D08G0528	Dt08: 5,919,667…5,922,528 −	402
	GrBASS3-2b	Gorai.004G059400.1	DD04: 5,751,294…5,754,644 −	411	GbBASS3-2Db	GOBAR_DD37325	Dt02: 36,780,966…36,783,829 −	412				
BASS4	GaBASS4	\	Scaffold2621: 42,711…66,814 −	407	GbBASS4A	GOBAR_AA02285	At12: 36,383,166…36,402,736 +	396	GhBASS4A	Gh_A12G2554	Scaffold3185_A12: 267,503…291,624 −	405
	GrBASS4	Gorai.008G099000.1	DD08: 28,310,229…28,330,759 +	407	GbBASS4D	GOBAR_DD30526	Dt12: 27,327,011…27,345,006 +	376	GhBASS4D	Gh_D12G0875	Dt12: 29,013,596…29,027,602 +	344
BASS5	GaBASS5	Cotton_A_07078	AA06: 74,981,927…74,984,601 −	435	GbBASS5A	GOBAR_AA03958	At12: 80,640,722…80,643,401 +	447	GhBASS5A	Gh_A12G2036	At12: 83,269,622…83,272,270 −	410
	GrBASS5a	Gorai.008G241200.1	DD08: 52,739,716…52,742,802 −	410	GbBASS5Da	GOBAR_DD30962	Dt12: 52,957,576…52,960,858 +	410	GhBASS5D	Gh_D12G2214	Dt12: 55,286,323…55,288,876 −	410
	GrBASS5b	Gorai.011G136100.1	DD11: 20,738,807…20,742,014 −	401	GbBASS5Db	GOBAR_DD00062	Dt10: 22,084,926…22,087,170 −	393				
	GrBASS5c	Gorai.003G059400.1	DD03: 10,283,519…10,286,466 −	335								

BASS family genes were identified from four cotton genomes using the HMMER software version 3.0. BASS, bile acid sodium symporter.

*Ga Gossypium arboretum*, *Gr Gossypium raimondii*, *Gb Gossypium barbadense, Gh Gossypium hirsutum*, *AA* Amino acid.

*Pseudogenes.

**Figure 1:**
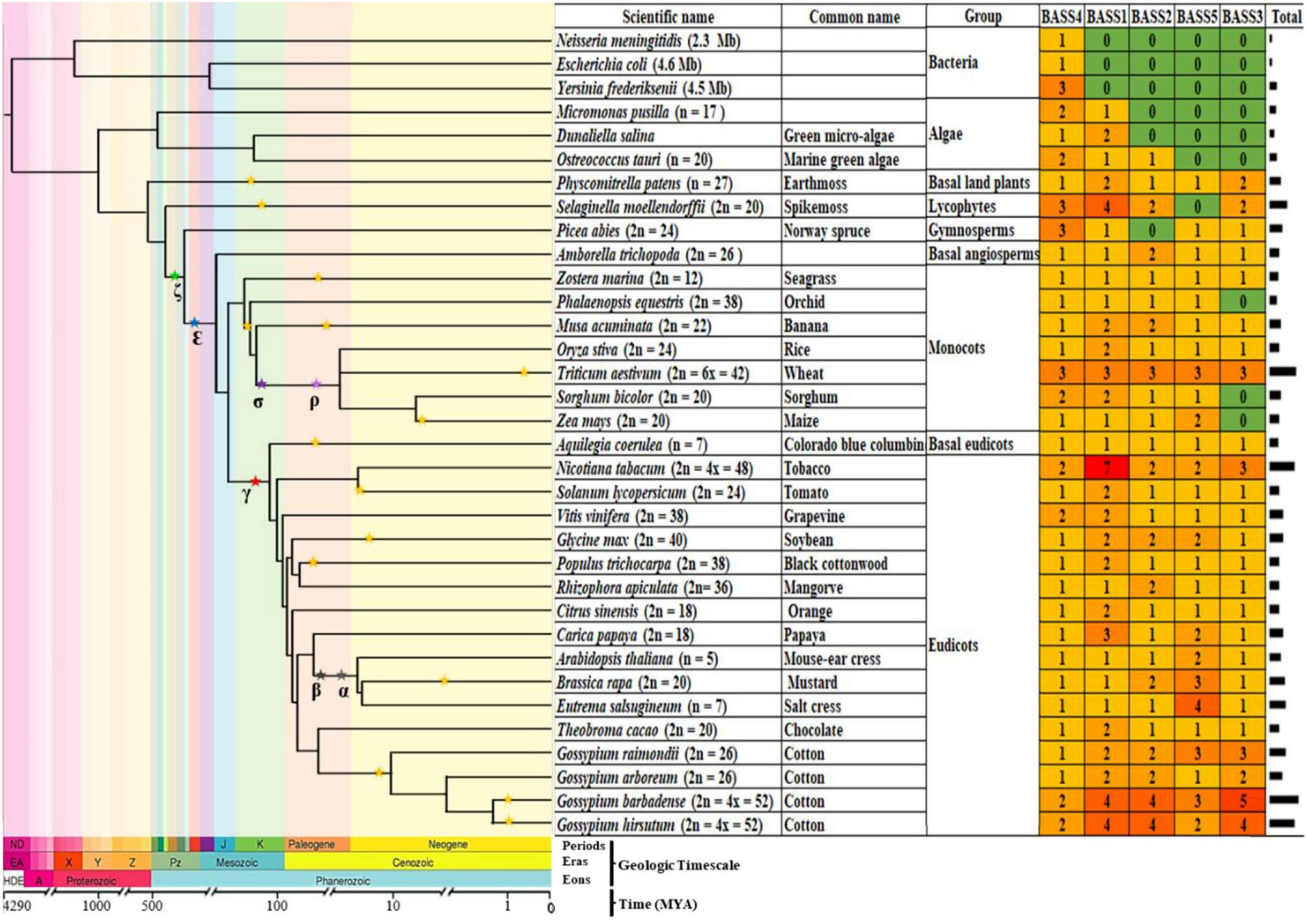
Species phylogeny and the number of BASS genes in each species. The species tree was inferred from Zeng et al.^21^. The divergence time was estimated by molecular clock dating from TimeTree^22^. Stars on the branches represent WGD events. **ζ**, ancestral seed plant WGD; ε, ancestral angiosperms WGD; γ, triplication event; β and α, two recent WGD events in Eudicots; σ and ρ, two polyploidy events in monocots. The scientific name, common name and group are followed by the number of BASSs identified. MYA, million years ago.

Additionally, we identified BASS genes from 30 other species including three bacteria, three algae, one basal land plants, one lycophytes, one gymnosperms and 21 angiosperms (Table S2). We found that BASS family genes were firmly maintained in all species from prokaryotes to eukaryotes selected in this study; however, the number of BASS members in prokaryotes and algae was less than those in embryophytes (Fig. 1). For example, there was only one BASS gene in two prokaryotic bacteria *Neisseria meningitidis* and *Escherichia coli,* but three BASS genes were identified in bacteria *Yersinia frederiksenii*. While there were three BASS genes in each of unicellular green algae *Micromonas pusilla* and *Dunaliella salina,* four BASS genes were observed in marine green algae *Ostreococcus tauri*.

**Figure 2:**
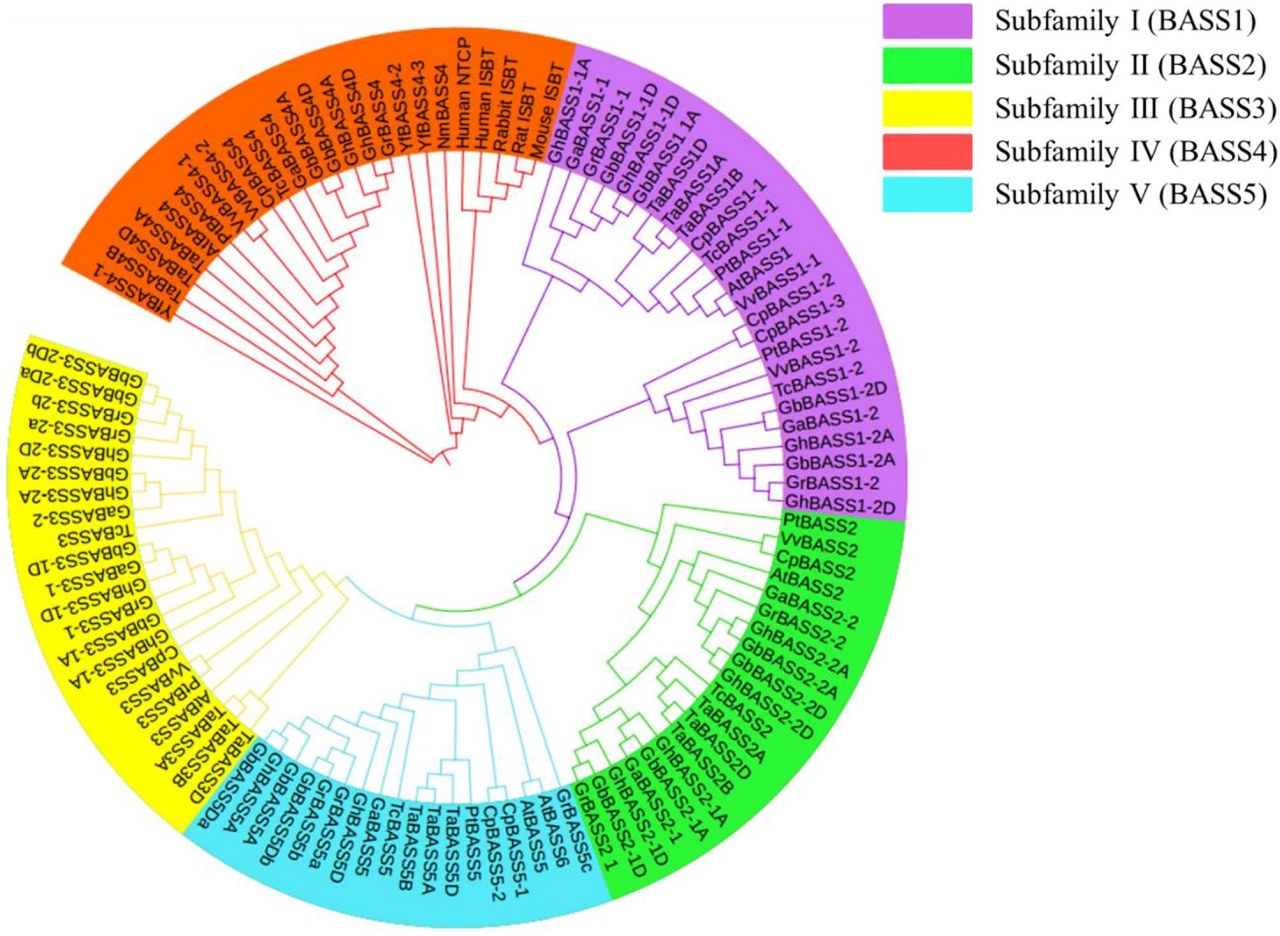
Phylogenetic tree of BASSs from plants, mammals and bacteria. The phylogenetic tree was constructed by employing the MAFFT program via the EMBL-EBI bioinformatics interface with default parameters, employing Gblocks to get conserved blocks, using PhyML to build the tree and visualizing the tree with the iTOL v4. *Ga Gossypium arboretum*, *Gr Gossypium raimondii*, *Gh Gossypium hirsutum*, *Gb Gossypium barbadense*, *Os Oryza sativa*, *At Arabidopsis thaliana*, *Tc Theobroma cacao*, *Vv Vitis vinifera*, *Pt Populus trichocarpa*, *Ta Triticum aestivum*, *Cp Carica papaya*, *Yf Yersinia frederiksenii*, *Nm Neisseria meningitides*, NTCP Sodium-taurocholate co-transporting polypeptide, *ISBT* Ileal sodium bile acid co-transporter.

To gain an insight into the evolutionary relationships of BASS genes, we aligned cotton protein sequences (53 genes) with 56 predicted BASS sequences from another 13 sequenced species including bacteria, plants and mammals for the construction of the phylogenetic tree. The BASSs could be classified into five subfamilies (Subfamily I to Subfamily V) according to the topology of the phylogenetic tree. All BASS1 genes belonged to subfamily I, BASS2 genes belonged to subfamily II, BASS3 genes belonged to subfamily III, BASS4 genes belonged to subfamily IV and BASS5 genes belonged to subfamily V, respectively (Fig. 2). Subfamily I, the BASS1 gene subfamily, stood as the largest group. The second-largest clade was the BASS4 gene subfamily, and BASS genes from bacteria and mammals were fallen into this group, which was followed by BASS3, BASS2 and BASS5 subfamily, respectively.

With respect to evolutionary relationships of BASS genes identified from different species, we found that members of the BASS4 subfamily were consistently retained in all selected species, even in prokaryotic bacteria that contained only BASS4 subfamily members (Figs. 1, 2). Intriguingly, fewer numbers of BASS4 subfamily genes were observed in almost tested angiosperm species. There were members of 2–3 BASS subfamilies in unicellular green algae, i.e., *Micromonas pusilla*, *Dunaliella salina* and *Ostreococcus tauri*. Members of 4 BASS subfamilies were discovered in basal land plants (*Physcomitrella patens*), lycophytes (*Selaginella moellendorffii*) and gymnosperms (*Picea abies*), while members of all subfamilies were kept in basal angiosperms (*Amborella trichopoda*). All tested dicotyledons and monocotyledons maintained all BASS subfamilies except for three C4 monocots (*Phalaenopsis equestris*, *Sorghum bicolor* and *Zea may*) in which there was a lack of the BASS3 subfamily gene. In general, the number of BASS genes was obviously larger in eudicots compared with monocots (Fig. 1). Hence, BASS family genes are very conserved starting from the prokaryotic bacteria and unicellular lower eukaryotic green algae to the multicellular angiosperm species. Collectively, BASS4 is assumed as the oldest gene, and this gene tends to be restored to the ‘singleton’ state in most tested angiosperm species by recently independent genome duplication events.

### Phylogenetic relationships, gene architectures and conserved motifs of cotton BASSs

**Figure 3:**
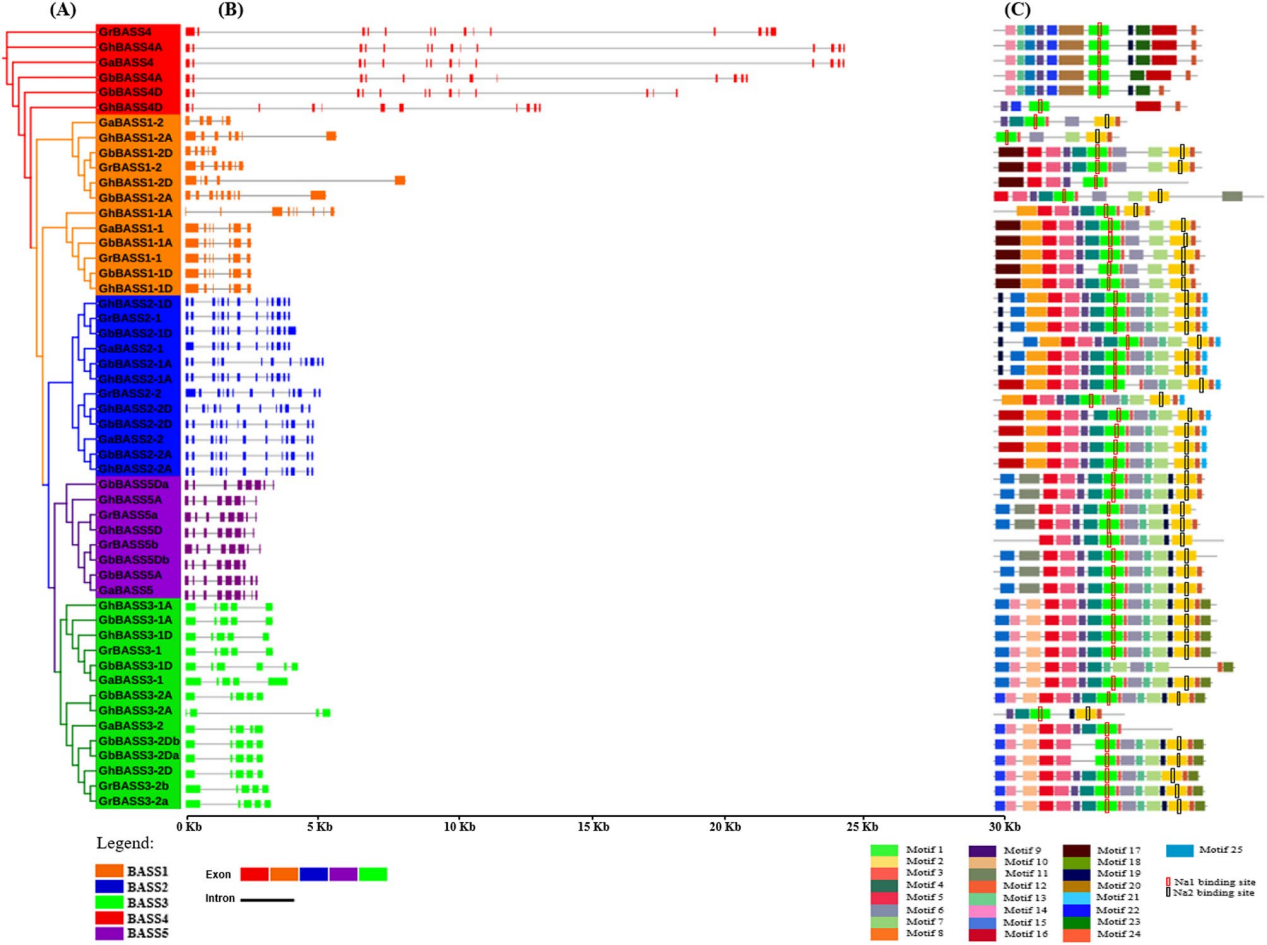
Phylogenetic relationships, gene architectures and conserved motifs of BASS genes from four *Gossypium* species. (**A**) Phylogenetic tree of putative cotton BASS family genes. The phylogenetic tree was constructed employing the MAFFT, Gblocks, PhyML and iTOL program. (**B**) Exon/intron organization of *Gossypium* BASS genes. Information for distribution of exons and introns was obtained from gff3 files of cotton genome annotation data, and gene architectures were depicted using the TBtools-JRE1.6 software. The color boxes indicate exons, and the grey lines represent introns. (**C**) Distributions of conserved motifs. Motifs were mined using the MEME software and depicted as 25 different color boxes. Na1 and Na2 binding sites are red and black rectangular boxes, respectively.

BASSs from four *Gossypium* species were tightly clustered in every subfamily. Moreover, BASSs from *Gossypium* species were closely clustered with those from *Theobroma cacao*, indicating that they shared the high sequence similarity and had close evolutionary relationships (Fig. 2). In addition, to detect the evolutionary relationships of BASSs in *G. arboreum*, *G. raimondii*, *G. barbadense* and *G. hirsutum*, the phylogenetic tree was also built using 53 cotton BASSs in which they were divided into five subfamilies too (Fig. 3A). In theory, one BASS gene in the diploid *G. arboreum* corresponds to one orthologous BASS gene in *G. raimondii* and two orthologous genes in the tetraploid *G. hirsutum* and *G. barbadense*. However, more BASS genes were found in *G. raimondii* (*GrBASS3-2b*, *GrBASS5b* and *GrBASS5c*) and *G. barbadense* (*GbBASS3-2b* and *GbBASS5b*). The BASS3 gene subfamily was composed of 14 members, each of BASS1 and BASS2 subfamilies consisted of 12 members, 9 members were grouped to form the BASS5 subfamily and the other 6 were for the BASS4 clade which served as an outgroup for other subfamilies (Fig. 3A).

In order to investigate gene structures of *Gossypium* BASS family genes, their exon/intron organizations were analyzed by mapping the coding sequences onto their genomic sequences. Overall, exon/intron structures of *Gossypium* BASS genes were relatively conserved within a subfamily, but they were quite diverse among subfamilies (Fig. 3B). For further details, BASS4 subfamily members owned the longest genomic sequences (14,006 to 24,121 bp) with 11–14 exons and comparatively longer introns. Within the BASS1 subfamily, there were two subgroups of which group one members kept the resembled formation of exons and introns except for *GhBASS1-1A*. On the other hand, group two was composed of genes with varied structural patterns of exon/intron, in which *GbBASS1-2D* carried the shortest genomic sequence (1145 bp) bearing 5 exons, leading to variation in the gene length of that group. The majority of participants in the BASS2 clade were comparatively less diverse in exon/intron structures, having 12 to 14 exons. Of the 9 BASSs in the BASS5 cluster, *GbBASS5Da* (8 exons) and *GbBASS5Db* (7 exons) were slightly different in exon/intron patterns, whereas the others preserved the resembled structure. With regard to the BASS3 clade, members showed similar exon/intron structure and gene length except for *GbBASS3-1D*, *GaBASS3-1* and *GhBASS3-2A* (Fig. 3B, Table S1). These results implied that numbers of exon/intron and structural organization of *Gossypium* BASS family genes are well correlated with the clades identified in the phylogenetic tree for each group.

To further clarify the structural feature of cotton BASS genes, the diversity of motif composition in 53 *Gossypium* BASS proteins was assayed by the MEME software. A total of 25 motifs designated as motif 1 to motif 25 were examined. Generally speaking, both of motif numbers and their distribution patterns in BASSs within a clade were fairly conservative (Fig. 3C). Motif numbers and patterns in the genes of the BASS1 clade were relatively varied, and there were 13 motifs of which motif 17 appeared as a unique motif of those genes belonged to this clade.11 motifs were found in the genes existed in the BASS4 cluster, and motif 15, 20 and 23 were unique for the genes of that cluster. The numbers of motifs in the members of the BASS2 and BASS5 subfamily were 16 and 14; motif 21 was exclusive to the members of the BASS2 subfamily, while motif 11 emerged only in genes of the BASS5 subfamily. The largest numbers of motifs (17 motifs) were produced by the genes involved in the BASS3 subfamily, and motif 10 and 18 were entirely restricted to these genes. Motif 1 was ubiquitous in every gene across all subfamilies, and motif 2 stood as a common motif except for the genes of the BASS4 subfamily (Fig. 3C). These results suggested that the similarity of gene structure and motifs distribution in each BASS subfamily provides evidence for the effective analysis of the phylogenetic tree.

### Chromosomal location, gene duplication and evolutionary relationships of *Gossypium* BASS family genes

Chromosomes were arranged as described by Wang et al.^23^ in the present study. With the exception of *GaBASS4* (scaffold2621), 7 other *GaBASSs* were distributed on 7 chromosomes (chromosome 1, 3, 6, 7, 8, 12 and 13) with the manner of one chromosome by one gene. *GrBASS* genes were mapped to 9 chromosomes (chromosome 1, 2, 3, 4, 8, 10, 11, 12 and 13) of which each of the chromosome 4 and 8 harbored 2 genes. *GhBASSs* were anchored on the chromosome A01, A04, A06, A07, A08, A12, A13, D01, D04, D06, D07, D08, D12 and D13, and every other chromosome except A12 and D12 which had two BASSs received one gene. Apart from *GbBASS3-2Db* (D02) and *GbBASS5Db* (D10), the chromosomal localization of 16 other *G. barbadense* BASSs was similar to the localization of orthologous counterpart genes of *G. hirsutum* (Tables 1, S1).

**Figure 4:**
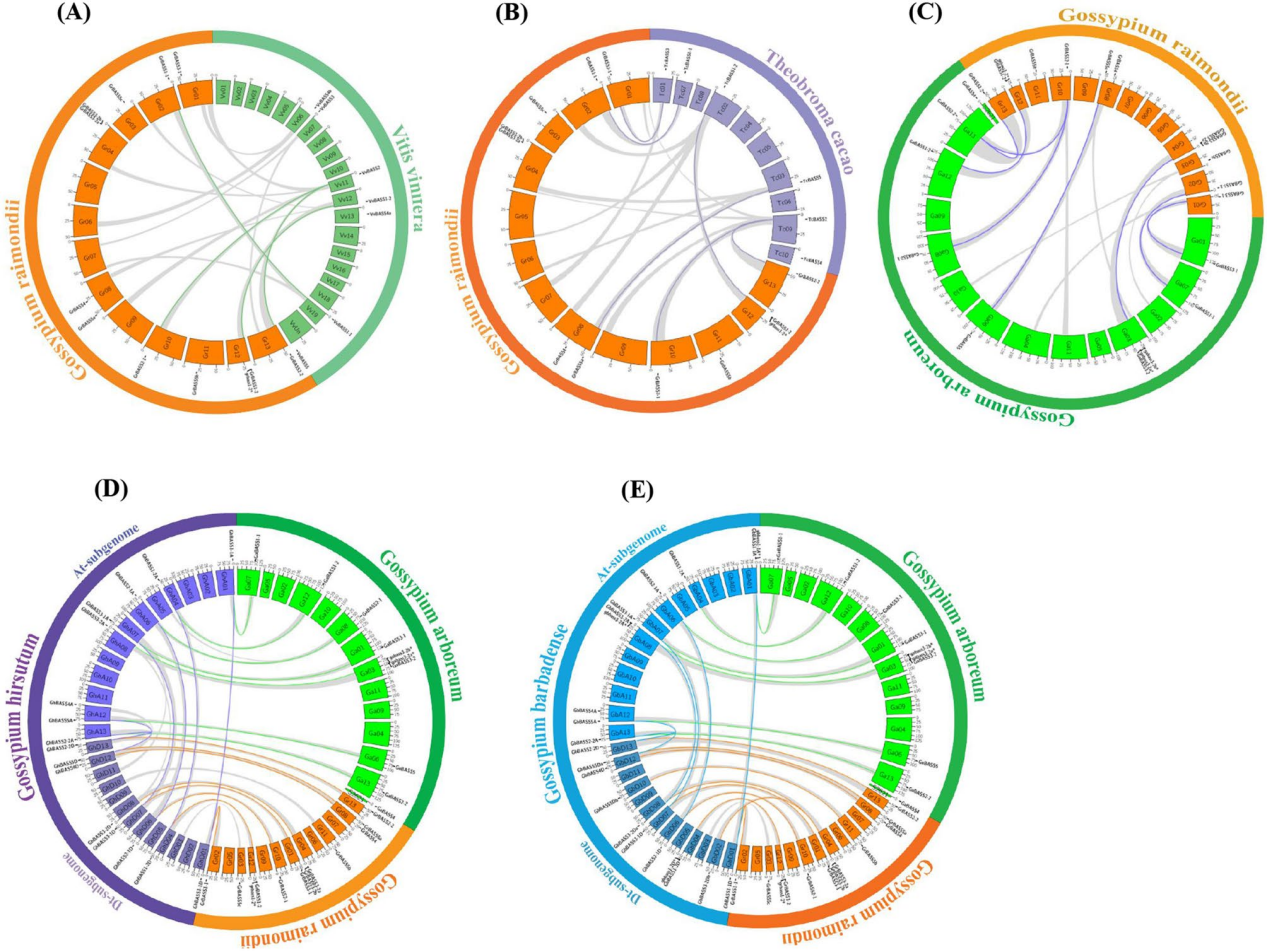
Genome-wide synteny analysis of BASS genes employing the MCScanX software. (**A**) Synteny analysis between *G. raimondii* and *V. vinifera.* Orange and green colored bars were depicted as chromosomes of *G. raimondii* (Gr01–13) and *V. vinifera* (Vv01–19 and VvUn), respectively. Green lines link orthologous gene pairs between *G. raimondii* and *V. vinifera.* (**B**) Synteny analysis between *G. raimondii* and *T. cacao*. Chromosomes of *G. raimondii* (Gr01–13) and *T. cacao* (Tc01–10) were filled with orange and purple, respectively. Purple lines connect orthologous gene pairs between *T. cacao* and *G. raimondii.* (**C**) Synteny analysis between *G. arboreum* and *G. raimondii*. Chromosomes of *G. raimondii* (Gr01–13) and *G. arboreum* (Ga01–13 and scaffold2621) were filled with orange and green, respectively. Blue lines bridge orthologous gene pairs between *G. arboreum* and *G. raimondii.* (**D**) Synteny analysis between *G. hirsutum* and two diploid species (*G. arboreum* and *G. raimondii*)*.* Purple, orange and green colored bars were depicted as chromosomes of *G. hirsutum* (GhA01–13 and GhD01–13), *G. raimondii* (Gr01–13) and *G. arboreum* (Ga01–13 and scaffold2621), respectively. Green lines link orthologous gene pairs between *G. arboreum* and *G. hirsutum,* orange lines connect orthologous gene pairs between *G. raimondii* and *G. hirsutum,* and purple lines bridge homoeologous gene pairs between At- and Dt-subgenome of *G. hirsutum.* (**E**) Synteny analysis between *G. barbadense* and two diploid species (*G. arboreum* and *G. raimondii*)*.* Blue, orange and green colored bars were depicted as chromosomes of *G. barbadense* (GbA01–13 and GbD01–13), *G. raimondii* (Gr01–13) and *G. arboreum* (Ga01–13 and scaffold2621), respectively. Green lines link orthologous gene pairs between *G. arboreum* and *G. barbadense,* orange lines connect orthologous gene pairs between *G. raimondii* and *G. barbadense*, and blue lines bridge homoeologous gene pairs between At- and Dt-subgenome of *G. barbadense.* Grey lines represent conserved synteny blocks between two different genomes and/or subgenomes of species. Putative BASS family genes were anchored to their corresponding chromosomes, and the symbol (*) means pseudogenes.

Whole-genome duplication (WGD) is an event which results in additional copies of the entire genome, which is also known as polyploidization^24^. If two or more paralogous genes were adjacent to each other on the same chromosome with no more than one intervening gene, they were defined as tandemly duplicated genes, whereas gene duplications between different chromosomes were designated as segmental duplications^25^. Following the criteria for inferring a gene duplication event^26^, we identified 3 duplicated gene pairs (*GaBASS1-1*/*GaBASS1-2*, *GaBASS2-1*/*GaBASS2-2* and *GaBASS3-1*/*GaBASS3-2*) from *G. arboretum*, while 2 duplicated gene pairs (*GrBASS1-1*/*GrBASS1-2* and *GrBASS2-1*/*GrBASS2-2*) and 2 triplicated gene pairs (*GrBASS3-1*/*GrBASS3-2a*/*GrBASS3-2b* and *GrBASS5a*/*GrBASS5b*/*GrBASS5c*) were found in *G. raimondii*. In tetraploid *Gossypium* species, BASS1, BASS2 and BASS3 genes were tetraplicate; however, there were 2 copies for BASS4 and BASS5 in *G. hirsutum*, as well as 2 copies for BASS4 and 3 copies for BASS5 in *G. barbadense*, respectively. Of the 53 genes from 4 *Gossypium* spp., only 2 genes were tandemly duplicated (*GrBASS3-2a*/*GrBASS3-2b*), whereas the others were derived from the segmental duplication. Besides, whole-genome duplications (WGD) resulted in more gene numbers of tetraploid *Gossypium* species, demonstrating its significant contribution to gene family expansion (Table 1, Fig. 4).

To further explore the genetic origins and evolutionary relationships of BASSs in cotton, we carried out the synteny analysis between the *G. raimondii* and *Vitis vinifera* genome, between the *G. raimondii* and *Theobroma cacao* genome, between the *G. raimondii* and *G. arboreum* genome, between the genome of *G. arboreum* and At-subgenomes of cultivated tetraploid cotton and between the genome of *G. raimondii* and Dt-subgenomes of cultivated tetraploid cotton. While *G. raimondii* vs. *V. vinifera* shared 14 inter-species syntenic regions harboring 4 orthologous gene pairs, 6 orthologous gene pairs were distributed in 16 inter-species syntenic regions of *G. raimondii* vs. *T. cacao* (Fig. 4A,B). 13 collinearity blocks with 8 orthologous gene pairs were detected between the *G. raimondii* and *G. arboreum* genome (Fig. 4C). Next, there were 8 syntenic blocks with 7 orthologous gene pairs between *G. arboreum* and At-subgenome of *G. hirsutum*, 9 blocks having 8 orthologous gene pairs between *G. raimondii* and Dt-subgenome of *G. hirsutum* and 9 blocks bearing 6 homoeologous gene pairs between At- and Dt-subgenome of *G. hirsutum* (Fig. 4D). Additionally, a sum of 23 collinearity blocks was identified between the diploid cotton and *G. barbadense*. Among them, two blocks individually possessed two orthologous gene pairs, but one harbored no gene pair. In contrast, the other collinearity blocks individually contained one gene pair (Fig. 4E). Based on these results, the analysis of syntenic blocks carrying orthologous or homoeologous genes showed important evidence to verify the evolutionary relationships of BASS genes in cotton.

To better understand the divergence and selection pressure of *Gossypium* BASS genes after polyploidization, values of nonsynonymous to synonymous substitution ratios (Ka/Ks) were calculated for homologous and homoeologous gene pairs among *Gossypium* species. Generally, Ka/Ks values of homologous and homoeologous BASS gene pairs between genomes and/or subgenomes were less than 1, in spite of the fact that the average Ka/Ks values of homologous gene pairs between the genome of *G. arboreum* and Dt-subgenome of *G. hirsutum* was 1.004, as well as Dt- and Dt-subgenome of *G. hirsutum* was 1.09. The total number of homologous and homoeologous gene pairs in the present study were 115, and the average Ka/Ks value of all 115 gene pairs was 0.88, which was less than 1 (Fig. S3). These data indicated that purifying selection was the main selective force operating on *Gossypium* BASS genes across species evolution.

### Cloning, subcellular localization and expression profiles of *GhBASS* genes

Based on the bioinformatics analyses, sequences of *GhBASS1* (Gene ID: Gh_D01G0342), *GhBASS2* (Gene ID: Gh_D06G0564), *GhBASS3* (Gene ID: Gh_D07G1990), *GhBASS4* (Gene ID: Gh_D12G0875) and *GhBASS5* (Gene ID: Gh_D12G2214) were retrieved for designing gene-specific primers (Table S3) in order to clone *GhBASS* genes from cotton cv. Zhong G5. We ultimately obtained 5 *GhBASS* cDNA sequences with the complete ORFs, and the cloned *GhBASSs* exhibited 99.5 to 99.89% identities with their corresponding query sequences derived from bioinformatics studies (Fig. S4). All of them maintained the conserved domains (SBF and bass domain), ten transmembrane regions and two Na^+^-binding sites, which are the characteristics of BASS family members.

**Figure 5:**
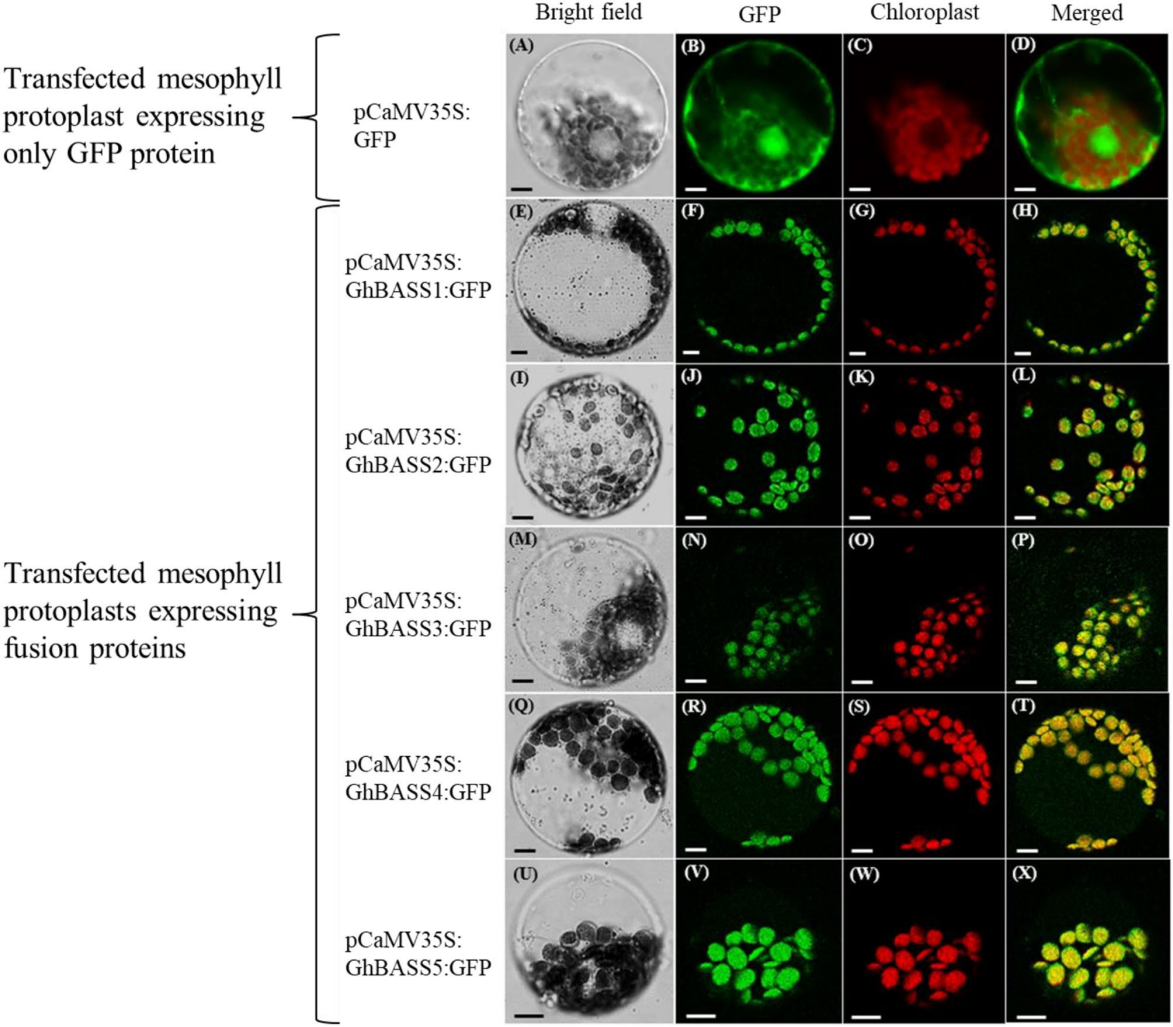
Subcellular localization of GhBASSs proteins. Recombinant plasmids bearing the fusion proteins (pCaMV35S:GhBASSs:GFP) and the empty plasmid bearing only the GFP protein (pCaMV35S:GFP) were independently transformed into *A. thaliana* mesophyll cells by the polyethylene glycol (PEG) transfection method^27^. All images were observed with a confocal laser scanning microscope. (**A**–**D**) *Arabidopsis* mesophyll protoplast expressing pCaMV35S:GFP protein; (**E**–**H**) *Arabidopsis* mesophyll protoplast expressing pCaMV35S:GhBASS1:GFP fusion protein; (**I**–**L**) *Arabidopsis* mesophyll protoplast expressing pCaMV35S:GhBASS2:GFP fusion protein; (**M**–**P**) *Arabidopsis* mesophyll protoplast expressing pCaMV35S:GhBASS3:GFP fusion protein; (**Q**–**T**) *Arabidopsis* mesophyll protoplast expressing pCaMV35S:GhBASS4:GFP fusion protein; (**U**–**X**) *Arabidopsis* mesophyll protoplast expressing pCaMV35S:GhBASS5:GFP fusion protein. Bars, 20 µm.

According to the data from The Arabidopsis Information Resource (TAIR), *Arabidopsis* BASSs are predicted to localize in the chloroplast envelope. Consistently, the chloroplast localization of GhBASSs was further predicted by web server predictors such as ChloroP (http://www.cbs.dtu.dk/services/ChloroP/) and LOCALIZER (http://localizer.csiro.au/). To prove this bioinformatics information, GhBASSs-GFP-transformed protoplasts and GFP-transformed protoplasts which served as a control were observed under a confocal laser scanning microscope. While observing GhBASSs-GFP-transfected protoplasts, GhBASSs-GFP signal was targeted only to the chloroplast membrane. On the other hand, as examining GFP-transfected protoplasts, GFP fluorescence was universally distributed throughout the cell, including in the nucleus (Fig. 5). This provided evidence that GhBASSs were localized in the chloroplast envelope.

**Figure 6:**
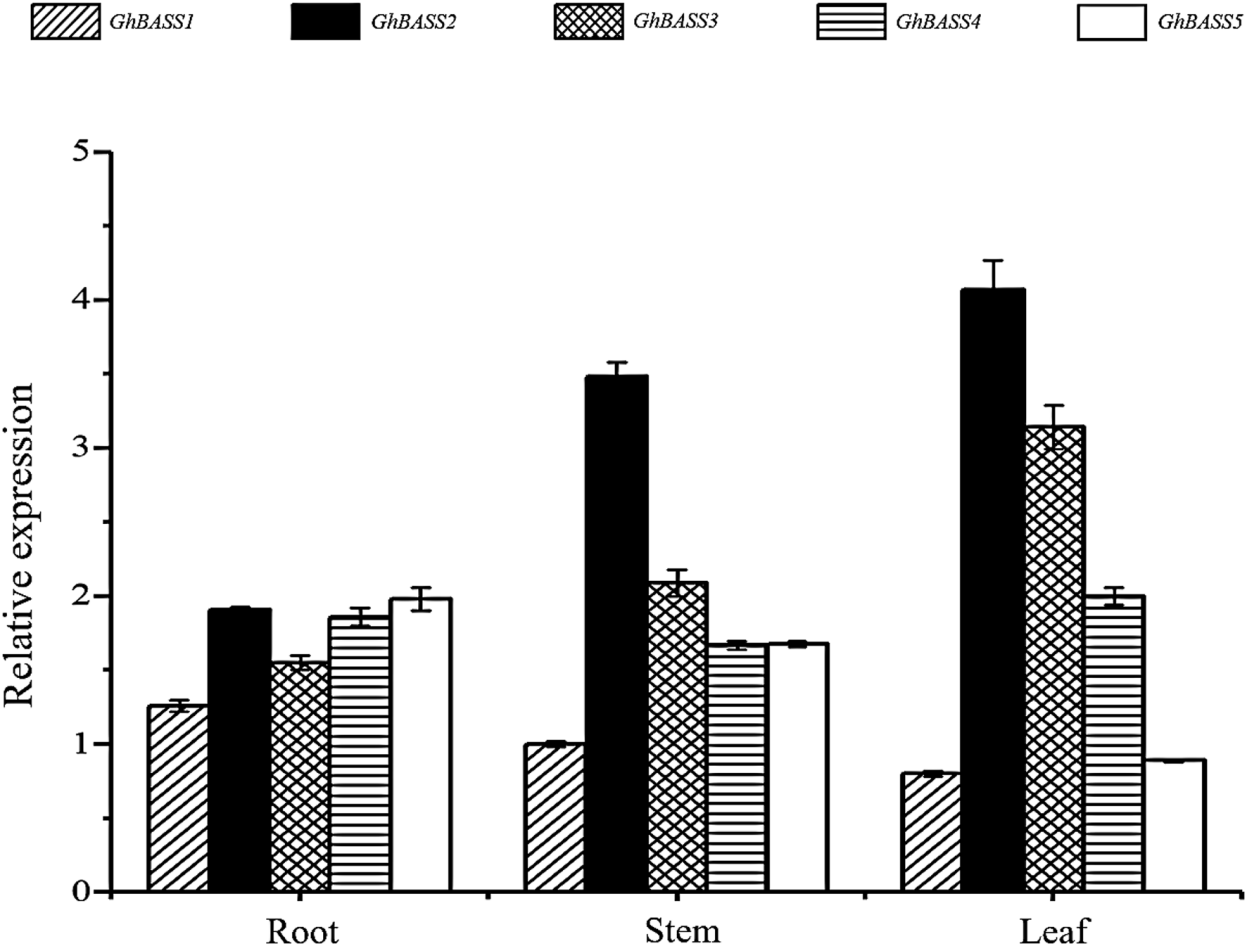
Expression patterns of *GhBASSs* in different tissues of cotton. Transcript levels were analyzed by qPCR and normalized to the *GhUBQ7* gene (GenBank accession no. DQ116441). Means and standard errors were based on three idependent biological replicates. Error bars indicate standard error (SE).

In order to examine the potential functions of *GhBASS* genes, their transcriptional levels in different tissues (root, stem and leaf) were monitored by qPCR with gene-specific primers (Table S3). Of these, the transcripts of *GhBASS2* and *GhBASS3* were expressed preferentially in leaf tissues; however, *GhBASS1* and *GhBASS5* showed higher expression levels in roots, and *GhBASS4* exhibited ubiquitous expression with no specific pattern (Fig. 6). These results indicated that BASS family members showed diverse expression patterns in different tissues.

**Figure 7:**
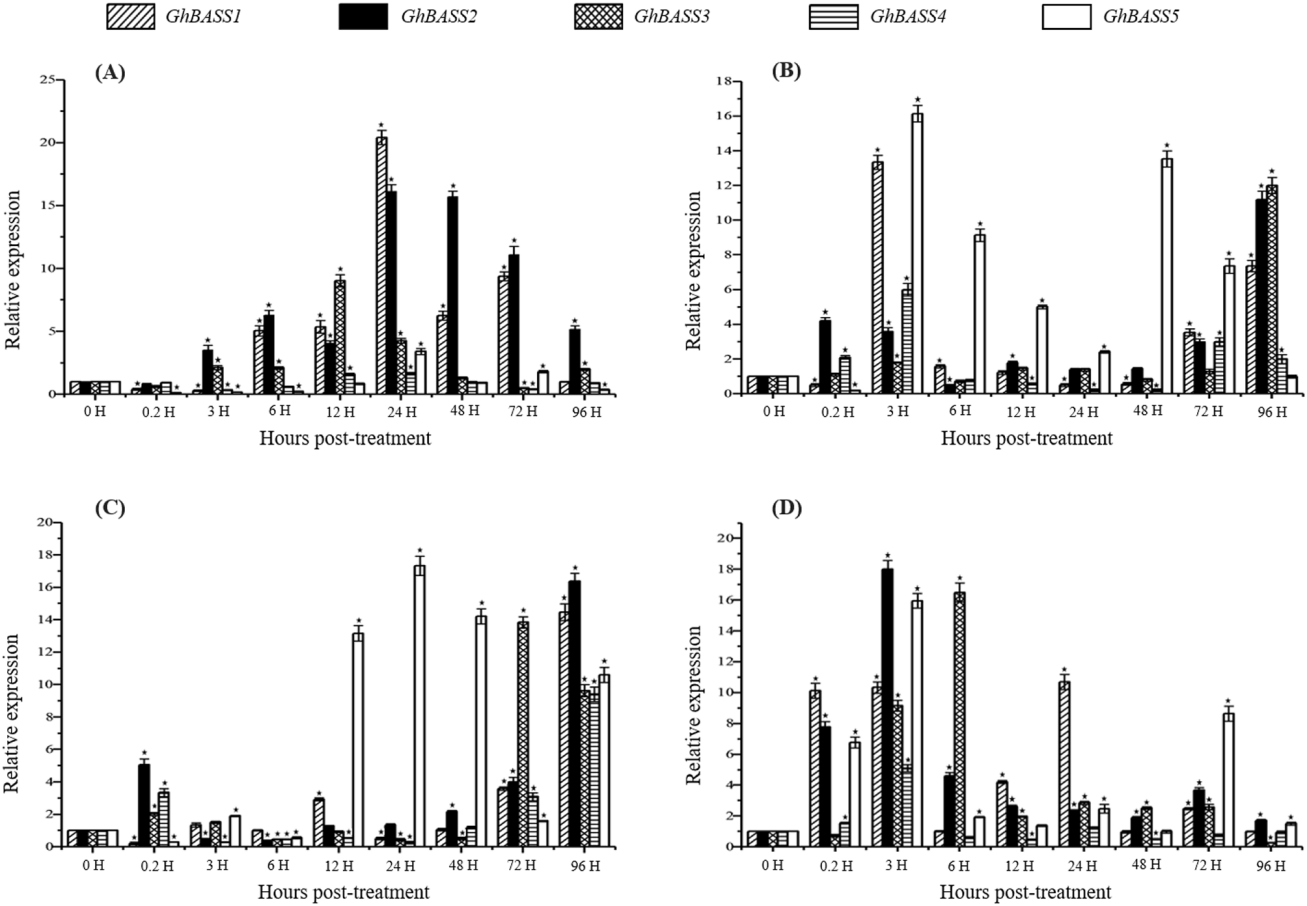
Expression patterns of *GhBASSs* under different abiotic stresses. (**A**) Expression levels of *GhBASSs* in cotton roots responded to the salt (NaCl) treatment. (**B**) Expression profiles of *GhBASSs* in cotton roots after treating with drought (PEG6000). (**C**) Transcript levels of *GhBASSs* in cotton leaves against heat stress (37 °C). (**D**) Expression patterns of *GhBASSs* in cotton leaves exposed to cold stress (4 °C). Expression levels were analyzed by qPCR and normalized to the *GhUBQ7* gene (GenBank accession no. DQ116441). Means and standard deviations were based on three independent biological replicates. Error bars represent the variation among three independent biological replications. Asterisks indicate the significant difference (*, *P* = 0.05) from 0 h post-treatment (hpt) by Tukey’s HSD test.

To determine whether BASS genes were induced by different stressors, we analyzed the expression patterns of *GhBASSs* during abiotic stresses, i.e., cold, heat, salt and drought. In response to NaCl treatment, *GhBASS1*, *GhBASS2* and *GhBASS3* were significantly induced; when *GhBASS1* and *GhBASS2* peaked at 24 h post-treatment (hpt), *GhBASS3* reached the maximum point at 12 hpt. By contrast, the transcriptional levels of *GhBASS4* and *GhBASS5* were decreased in responding to salt except at a few time points (Fig. 7A). Generally speaking, *GhBASSs* exhibited up-regulation after the drought induction, of which *GhBASS5* was greatly induced, while the other genes were moderately induced (Fig. 7B). When exposed to heat stress, *GhBASS5* was clearly up-regulated, whereas the transcript level of *GhBASS1* was increased only at 3, 12, 72 and 96 hpt. The expression of *GhBASS2*, *GhBASS3* and *GhBASS4* was increased as early as 0.2 hpt and then rapidly fell until 72 and 96 hpt during heat stress (Fig. 7C). Overall, the transcripts of *GhBASS1*, *GhBASS2*, *GhBASS3* and *GhBASS5* were elevated after the cold treatment; in contrast, *GhBASS4* was expressed only at 0.2, 3 and 24 hpt (Figs. 7D, S6). These results suggested that the expression patterns of *G. hirsutum* BASS genes were widely varied in response to different abiotic stresses.

**Figure 8:**
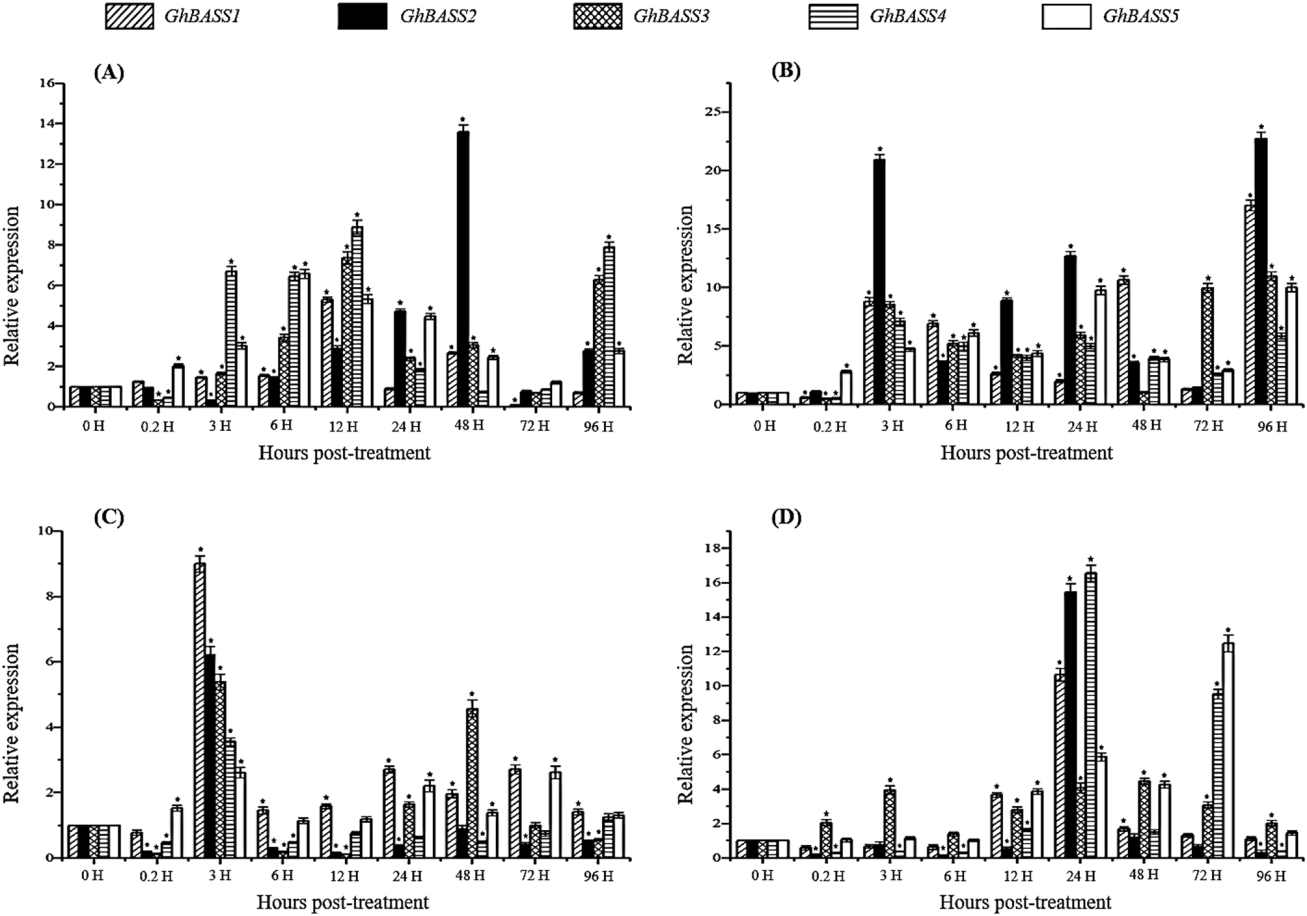
Expression patterns of *GhBASSs* during the treatments of various phytohormones. (**A**) Expression levels of *GhBASSs* in cotton leaves responded to the methyl viologen (MV) treatment. (**B**) Expression profiles of *GhBASSs* in cotton leaves after treated with abscisic acid (ABA). (**C**) Transcript levels of *GhBASSs* in cotton leaves against salicylic acids (SA). (**D**) Expression patterns of *GhBASSs* in cotton leaves exposed to gibberellic acids (GA3). Expression levels were analyzed by qPCR and normalized to the *GhUBQ7* gene (GenBank accession no. DQ116441). Means and standard deviations were based on three independent biological replicates. Error bars represent the variation among three independent biological replications. Asterisks indicate the significant difference (*, *P* = 0.05) from 0 hpt by Tukey’s HSD test.

Next, we investigated the responses of *GhBASS* genes against the treatments of different phytohormones such as salicylic acid (SA), abscisic acid (ABA) and gibberellic acid (GA3), as well as methyl viologen (MV) which is the herbicide and experimental tool, and induces the generation of reactive oxygen species (ROS) in the chloroplast^28^. All *G. hirsutum* BASS genes were induced and accumulated at higher levels after MV and ABA treatments except at some time points. With the influence of these two inducers, the transcriptional abundance of *GhBASS2* was the highest among the tested genes (Fig. 8A,B). Under SA conditions, transcript levels of *GhBASS1* and *GhBASS5* were significantly elevated, reaching their highest levels at 3 and 72 hpt, respectively, meanwhile those of *GhBASS2*, *GhBASS3* and *GhBASS4* were not clear with the fluctuated expression patterns (Fig. 8C). In response to the exogenous GA3 application, *GhBASS3* and *GhBASS5* were highly expressed; *GhBASS3* reached its peak at 48 hpt, and *GhBASS5* peaked at 72 hpt. *GhBASS2* was dramatically expressed at 24 hpt, but its expression was diminished at other time points in relation to GA3. *GhBASS1* and *GhBASS4* showed oscillated expression levels reaching their peaks at 24 hpt when subjected to GA3 (Figs. 8D, S7). As a whole, this finding revealed that all candidate genes were induced by at least two of four inducers, implying that they might play important roles in signaling pathways.

### *GhBASSs* knock-down plants show varied levels of salt tolerance

**Figure 9:**
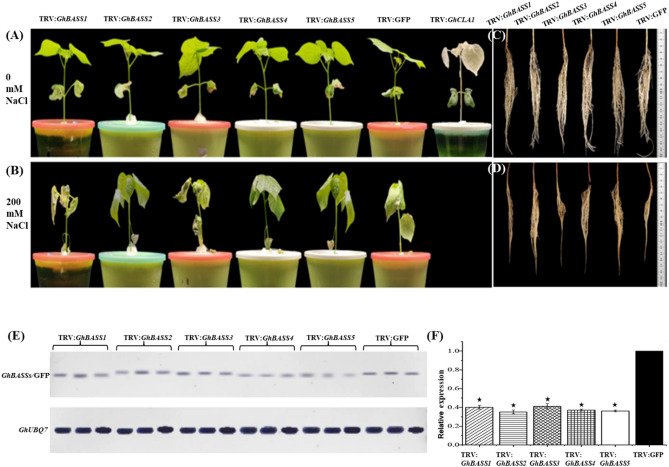
Varied salt-tolerant levels of *GhBASSs* knock-down plants by *Agrobacterium*-mediated VIGS. Shoot performance of TRV:*GhBASSs* and TRV:GFP before exposed to NaCl (**A**) and after subjected to 200 mM NaCl for 5 days (**B**). Root performance of TRV:*GhBASSs* and TRV:GFP before treated with NaCl (**C**) and after treated with 200 mM NaCl for 5 days (**D**). Related transcript levels of *GhBASSs* in *GhBASSs* knock-down plants by RT-PCR (**E**) and qPCR (**F**). *GhUBQ7* (GenBank accession no. DQ116441) was used as an internal control. Means and standard deviations were based on three independent biological replicates. Error bars represent the variation among three independent biological replications. Asterisks indicate the significant difference (*, *P* = 0.05) from the control (TRV:GFP) by Tukey’s HSD test.

Virus-induced gene silencing (VIGS) is increasingly used for rapid and large-scale gene analyses in functional genomics, and the tobacco rattle virus (TRV) is a powerful vector for VIGS in *Gossypium* species^29^. *Agrobacterium*-mediated VIGS has been successfully employed in a wide range of dicotyledonous and monocotyledonous species for various functional studies^30^. With the application of VIGS, a more detailed study on the functions of *GhBASSs* in relation to salt stress was performed. *GhCLA1* is a homologous gene to DXS1/CLA1 in *A*. *thaliana*. The *Arabidopsis* Cloroplastos alterados 1 gene (*AtCLA1*) involved in chloroplast development^31^, and knocking-out *AtCLA1* resulted in an albino phenotype in *Arabidopsis*^32^, which provides an excellent visual marker for silencing efficiency. Each of the TRV-based VIGS vectors was specifically constructed targeting to the non-conserved regions of *GhBASS* genes and outside the conserved domains to avoid interference with other BASS genes and other SBF proteins. Two weeks after agroinfiltration, TRV:*GhCLA1* exhibited a highly uniformed photobleaching symptom in newly emerged leaves, meanwhile non-symptomatic new leaves were observed in TRV:GFP plants under the same conditions, indicating that silencing of *GhCLA1* occurred specifically in TRV:*GhCLA1* infected plants (Fig. 9A). Furthermore, RT-PCR and qPCR were employed to verify the gene silencing efficiency by examining the expression levels of endogenous BASS genes. The transcript abundances of *GhBASSs* in TRV:*GhBASSs* plants were obviously diminished compared with those in TRV:GFP plants (Figs. 9E,F, S8). These results provided evidence that the *Agrobacterium*-mediated virus-induced gene silencing system correctly functioned and could be used in further experiments.

**Figure 10:**
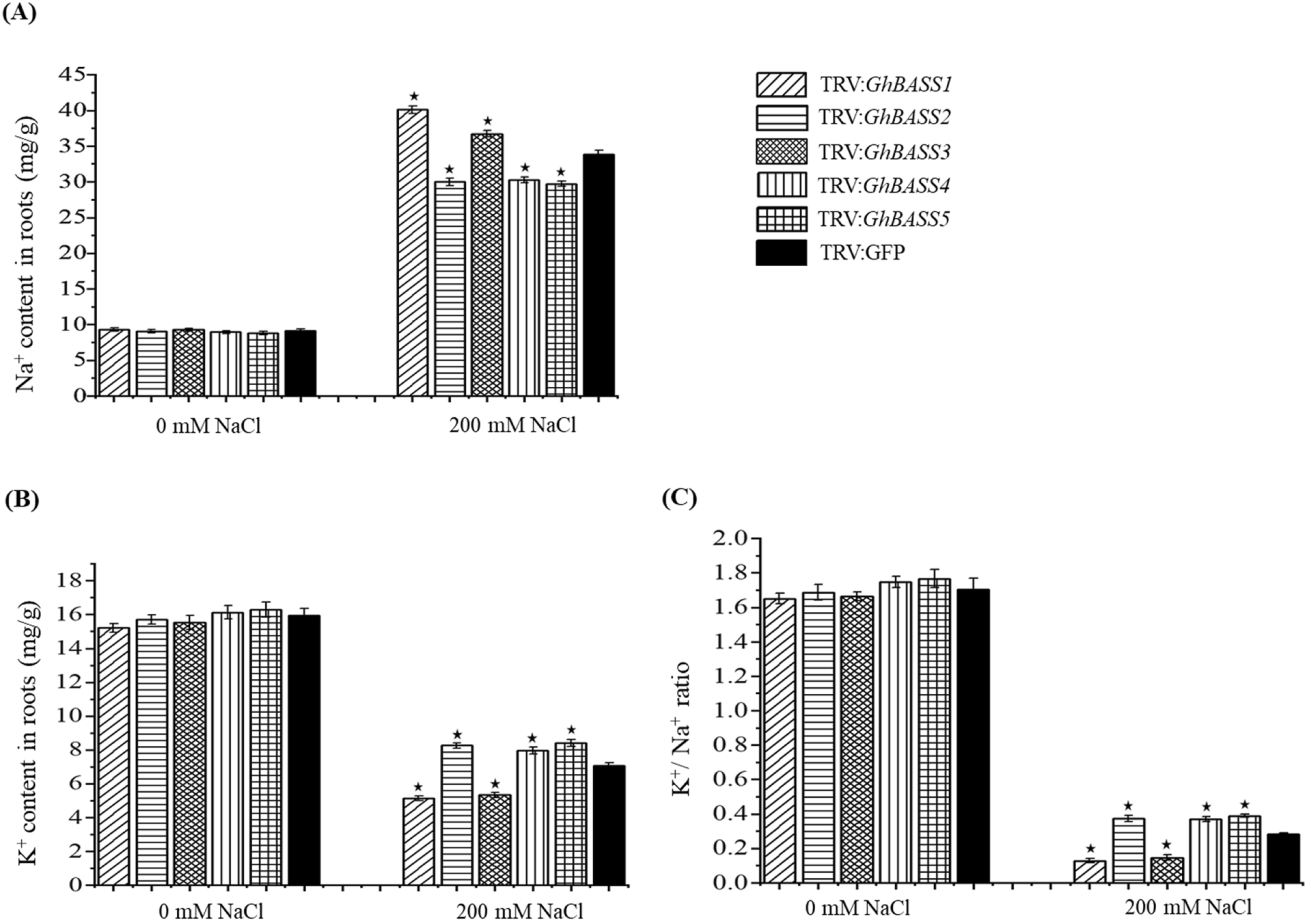
Ion contents in *GhBASSs* knock-down roots with the absence or presence of 200 mM NaCl for 5 days. (**A**) Na^+^ content in the roots of gene silencing plants. (**B**) K^+^ content in the roots of gene silencing plants. (**C**) K^+^/Na^+^ ratio. The ionic concentration is presented as mg/g dry weight. Means and standard deviations were based on three independent biological replicates. Error bars represent the variation among three independent biological replications. Asterisks indicate the significant difference (*, *P* = 0.05) from TRV:GFP by Tukey’s HSD test.

Even though TRV:*GhBASSs* and TRV:GFP plants showed the same growth performance during the non-stressed conditions (Fig. 9A), after treated with salt stress for 5 days, TRV:*GhBASS1* infiltrated plants revealed the most serious symptom of salinity stress having more wilting leaves, followed by TRV:*GhBASS3* infiltrated plants when compared with the control plants. On the other hand, TRV:*GhBASS2*, TRV:*GhBASS4* and TRV:*GhBASS5* infiltrated plants grew stronger with less salt-damaged leaves in comparison with the control plants under the same conditions (Fig. 9B). In addition, the root performance of TRV:*GhBASSs* was quite similar to that of TRV:GFP under normal conditions (Fig. 9C). Although roots of TRV:*GhBASS2*, TRV:*GhBASS4* and TRV:*GhBASS5* plants were stronger and bigger than those of the controls, TRV*:GhBASS1* and TRV*:GhBASS3* plants possessed weakened roots after they had been exposed to salinity conditions (Fig. 9D), which matched up to the shoot performance of those plants. To confirm this result, we analyzed Na^+^ and K^+^ contents in all tested plants. Ions accumulation in the tested plants were enormously different between before and after the 200 mM NaCl application. Na^+^ accumulation in *GhBASS2*, *GhBASS4* and *GhBASS5* knock-down plants was greatly lower, but in *GhBASS1* and *GhBASS3* knock-down plants was higher than those in the control plants after subjected to salt, in spite of the fact that their Na^+^ contents were likely the same under non-salt exposure (Fig. 10A). By contrast, the scenario of K^+^ accumulation was totally opposite to that of Na^+^ accumulation during salt stress. Under normal conditions, K^+^ content in all tested plant was not clearly different (Fig. 10B). Consequently, the K^+^/Na^+^ ratio was significantly declined in *GhBASS1* and *GhBASS3* knock-down plants, but distinctly raised in *GhBASS2*, *GhBASS4* and *GhBASS5* knock-down plants compared with that in the controls during salt treatment, even though the difference in this ratio was not clear under the mock conditions (Fig. 10C). According to these results, it could be concluded that *GhBASS1* and *GhBASS3* positively regulated, whereas *GhBASS2*, *GhBASS4* and *GhBASS5* played a negative regulatory role in salt tolerance of their corresponding knock-down plants.

### Overexpression of *GhBASS2 *and *GhBASS5* impairs salt tolerance of transgenic *Arabidopsis*

**Figure 11:**
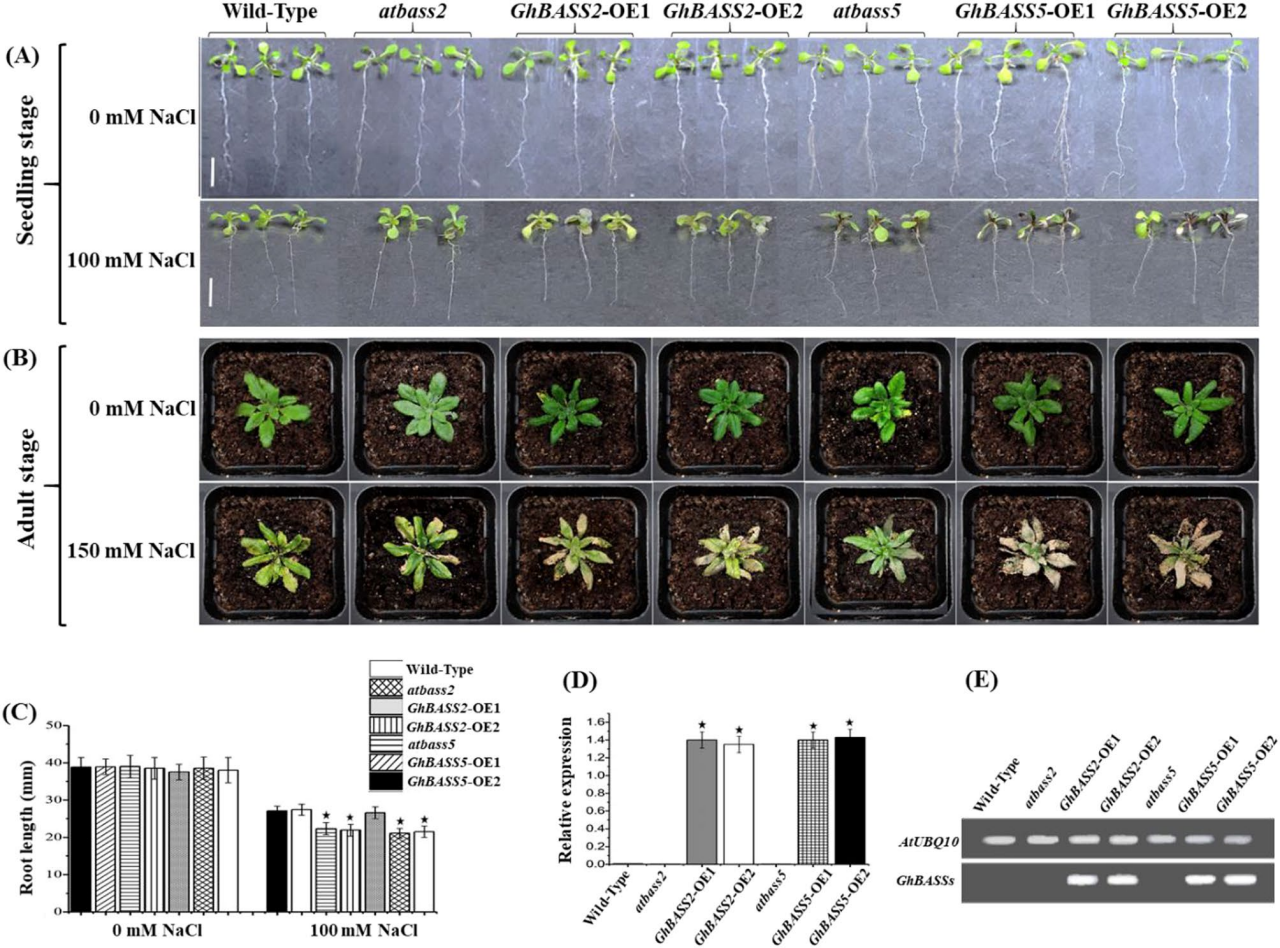
Constitutively overexpressing *GhBASS2* and *GhBASS5* weaken salt stress resistance in transgenic *Arabidopsis*. (**A**) The seedling’s phenotypes of transgenic OE lines, wild-type and loss-of-function mutants growing on the 1/2 MS medium supplemented with 0 or 100 mM NaCl for ten days. Bar = 10 mm. (**B**) The adult’s phenotypes of transgenic OE lines, wild-type and loss-of-function mutants after a seven-day treatment with 0 or 150 mM NaCl. (**C**) Root length of the wild-type, mutants and OE seedlings treated with 0 or 100 mM NaCl. (**D**, **E**) Transcript levels of *GhBASS2* and *GhBASS5* in wild-type, mutants and OE plants by qPCR and RT-PCR. The *AtUBQ10* gene (GenBank accession no. AT4G05320) was used as an internal standard. Means and standard deviations were based on three independent biological replicates. Error bars represent the variation among three independent biological replications. Asterisks indicate the significant difference (*, *P* = 0.05) from WT by Tukey’s HSD test.

Gene overexpression is a process which makes the abundant target protein expression substantially^33^. By this means, phenotypes resulted from overexpressing target genes can be observed, which allows the functional characterization of those genes^34^. *A. thaliana* (Col-0) was used to generate transgenic plants overexpressing selected *GhBASS* genes in the present study, because it grows quickly, produces many seeds and is genetically well characterized^35^. Therefore, we continued to conduct the overexpression experiment for *GhBASS2* and *GhBASS5* which were selected based on our previous findings and information on other literatures. With the nested sequence-specific and shorter arbitrary degenerate primers, thermal asymmetric interlaced PCR (TAIL-PCR) is a highly efficient method to characterize the genomic DNA regions flanking the T-DNA insertion sites, which is applicable for the initial screening of independent transgenic lines^36–38^. Thus, transgenic T1 seedlings surviving on the selective medium supplemented with 50 μg ml^−1^ hygromycin were validated by TAIL-PCR. In the TAIL-PCR gel electrophoresis results (Fig. S10A,B), WT control showed faint non-specific bands, whereas negative control didn’t reveal any band even in the tertiary TAIL-PCR products. Interestingly, only one clear and bright band was observed in each of the secondary TAIL-PCR products of OE lines, even thought there was no clear and bright band in the primary TAIL-PCR products. Consistently, clearer and brighter bands were found in the tertiary TAIL-PCR products, and the sizes of those bands were similar to the sizes of bands in the secondary TAIL-PCR products. Importantly, different molecular weight bands, which were clearly and brightly visible in the tertiary TAIL-PCR products, between different OE transgenic lines were observed, indicating different insertion sites of interest DNA sequences (*GhBASS2* and *GhBASS5* gene) into the genome of *A. thaliana* (Col-0)^36–38^. These gel results were corroborated by the flanking sequence tags by direct sequencing the tertiary TAIL-PCR products (Fig. S10C,D). Accordingly, these OE transgenic lines were independent with different T-DNA insertion loci in the *Arabidopsis* genome. After *GhBASS2*-OE and *GhBASS5*-OE plants had been validated as genuinely transgenic plants through the application of TAIL-PCR, qPCR and RT-PCR (Figs. 11D,E, S9–S10), the salt tolerance detection of transgenic plants was carried out. To determine the growth performance of transgenic seedlings, seeds of T3 homozygous lines (*GhBASS2*-OE1, *GhBASS2*-OE2, *GhBASS5*-OE1 and *GhBASS5*-OE2), loss-of-function mutants (*atbass2* and *atbass5*) and wild-type (WT) were grown on 1/2 MS medium with or without 100 mM NaCl. During salt stress conditions, OE lines exhibited much worse phenotypes with yellowing leaves and weakened roots in comparison with mutants and WT, in spite of the fact that their shoot and root performances were similar under normal situations (Fig. 11A,C). For further clarification of salt sensitivity of soil-grown *GhBASS2*-OE and *GhBASS5*-OE lines, 3-week-old WT, mutants and transgenic plants were irrigated with 150 mM NaCl. After 7 days of the salt treatment, *GhBASS2*-OE and *GhBASS5*-OE plants showed more impaired phenotypes with chlorotic and wilting leaves when compared with the control plants. Nonetheless, they had much better phenotypes displaying deeper green leaves than those of the controls once exposed to the non-salt treatment (Fig. 11B).

**Figure 12:**
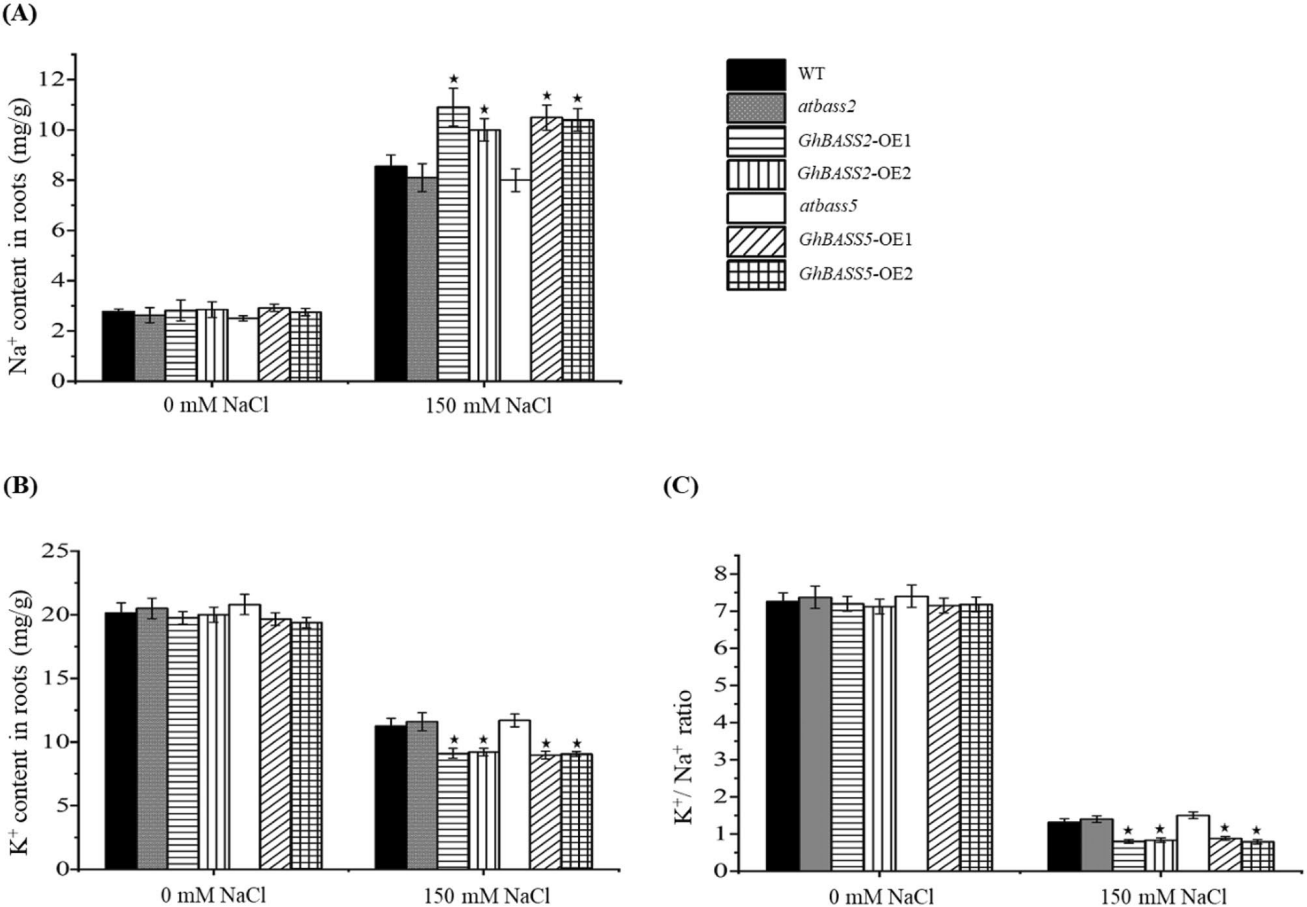
Ion contents in *GhBASS2*- and *GhBASS5*-overexpressed plants with the absence or presence of 150 mM NaCl for 7 days. (**A**) Na^+^ content in the roots of OE lines, mutants and WT plants. (**B**) K^+^ content in the roots of OE lines, mutants and WT plants. (**C**) K^+^/Na^+^ ratio. The ionic concentration is presented as mg/g dry weight. Means and standard deviations were based on three independent biological replicates. Error bars represent the variation among three independent biological replications. Asterisks indicate the significant difference (*, *P* = 0.05) from WT by Tukey’s HSD test.

To verify the phenotype results, a direct investigation of ion levels in tissues was demanded. Before NaCl treatment, Na^+^ contents of tested plants were not clearly different (WT = 2.77 ± 0.11 mg g^-1^, *atbass2* = 2.63 ± 0.31 mg g^-1^, *GhBASS2*-OE1 = 2.81 ± 0.41 mg g^-1^, *GhBASS2*-OE2 = 2.84 ± 0.31 mg g^-1^, *atbass5* = 2.5 ± 0.11 mg g^-1^, *GhBASS5*-OE1 = 2.91 ± 0.15 mg g^-1^ and *GhBASS5*-OE2 = 2.74 ± 0.15 mg g^-1^; Mean ± SD for n = 3). After 150 mM NaCl treatment, the mean values of Na^+^ contents of the tested plants were distinctly varied (8.54 ± 0.45 mg g^-1^ from WT, 8.1 ± 0.55 mg g^-1^ from *atbass2*, 10.9 ± 0.75 mg g^-1^ from *GhBASS2*-OE1, 10 ± 0.45 mg g^-1^ from *GhBASS2*-OE2, 8 ± 0.45 mg g^-1^ from *atbass5*, 10.49 ± 0.5 mg g^-1^ from *GhBASS5*-OE1 and 10.39 ± 0.46 mg g^-1^ from *GhBASS5*-OE2; Mean ± SD for n = 3) (Fig. 12A). On the other hand, the average K^+^ concentration of OE plants was always lower than those of WT and mutants under both conditions (WT = 20.14 ± 0.8 mg g^-1^, *atbass2* = 20.5 ± 0.8 mg g^-1^, *GhBASS2*-OE1 = 19.76 ± 0.51 mg g^-1^, *GhBASS2*-OE2 = 20 ± 0.6 mg g^-1^, *atbass5* = 20.8 ± 0.8 mg g^-1^, *GhBASS5*-OE1 = 19.66 ± 0.49 mg g^-^1 and *GhBASS5*-OE2 = 19.39 ± 0.41 mg g^-1^ for 0 mM NaCl; 11.26 ± 0.6 mg g^-1^ from WT, 11.6 ± 0.71 mg g^-1^ from *atbass2*, 9.1 ± 0.39 mg g^-1^ from *GhBASS2*-OE1, 9.2 ± 0.32 mg g^-1^ from *GhBASS2*-OE2, 11.7 ± 0.49 mg g^-1^ from *atbass5*, 8.96 ± 0.31 mg g^-1^ from *GhBASS5*-OE1 and 9.06 ± 0.22 mg g^-1^ from *GhBASS5*-OE2 for 150 mM NaCl; Mean ± SD for n = 3) (Fig. 12B). As a result, K^+^/Na^+^ ratio in the tissues of transgenic plants was significantly lower than those in the tissues of mutants and wild-type plants when allowed to salt-stressed conditions; however, this ratio was not distinctly varied among the tested plants under non-stressed conditions (Fig. 12C). These results indicated that the enhanced salt susceptibility in transgenic plants constitutively expressing *GhBASS2* and *GhBASS5* resulted from the increased Na^+^ accumulation.

## Discussion

### Genome-wide identification of the BASS gene family in cotton

Plants underwent a higher rate of gene duplication in comparison with other eukaryotes^39^, and gene and genome duplications are responsible for variability in gene numbers^40^; therefore, the number of BASS genes was greatly varied in different species (Fig. 1). The peculiar distribution of the BASS gene family suggests a plausible scenario for its origin that BASS4 emerged in bacteria, later green algae obtained BASS4, BASS1 and BASS2, and then land plants (bryophytes and vascular plants) possessed all BASS members (Fig. 1). Hence, all recent BASS genes were derived from an ancestral BASS4 gene which evolved before the divergence from the common ancestor of prokaryotes and eukaryotes, indicating its essential role in living organisms.

In our study, we identified 8, 11, 16 and 18 BASS genes from *G. arboreum*, *G. raimondii*, *G. hirsutum* and *G. barbadense*, respectively (Tables 1, S1, S2). Gene duplication contributing to the amplification of gene numbers is the key mechanism behind the expansion of a gene family^41^. Likewise, cotton BASSs experienced gene duplication events. Consequently, each of BASS1, BASS2 and BASS3 was duplicated at least two copies in diploids (*G. arboreum* and *G. raimondii*) and four copies in tetraploids (*G. hirsutum* and *G. barbadense*). Unlikely, BASS4 was resistant to expand its number or it tended to be restored to the ‘singleton’ state during polyploidization followed by diploidization events; thereby, the number of BASS4 is only one in diploids and two in tetraploids. Duplication pattern of BASS5 was varied among *Gossypium* spp. that there were three paralogues (*GrBASS5a*, *GrBASS5b* and *GrBASS5c*) in *G. raimondii*, two homeologs (*GhBASS5A* and *GhBASS5D*) in *G. hirsutum* and three homeologs (*GbBASS5A*, *GbBASS5Da* and *GbBASS5Db*) in *G. barbadense*, while only *GaBASS5* was anchored in *G. arboretum* (Tables 1, S1). Collectively, some BASS members have expanded their numbers, whereas other members are expansion-resistant, hinting the selective duplication of BASS genes in cotton^42^. Additionally, since only one paralogous gene pair (*GrBASS3-2a*/*GrBASS3-2b*) derived from tandem duplication, WGD as well as segmental duplication conferred the expansion of the BASS gene family in cotton.

Of the 16 *GhBASSs*, 8 homeologs were from the At-subgenome and another 8 homeologs were from the Dt-subgenome; hence, every gene had its corresponding ortholog in *G. arboretum* and *G. raimondii*. Likewise, *GbBASSs* individually possessed their orthologous counterpart genes of *G. arboretum* and *G. raimondii*. Nonetheless, neither corresponding orthologs of 3 *GrBASSs* (*GrBASS3-2b*, *GrBASS5b* and *GrBASS5c*) in the Dt-subgenome of *G. hirsutum* nor the corresponding ortholog of *GrBASS5c* in the Dt-subgenome of *G. barbadense* was discovered (Tables 1, S1), indicating that these genes were presumably lost in the cultivated tetraploid cotton during or after polyploidization events^42^.

For further details of gene losses in cotton, the study on evolutionary relationships of BASS family members from *Gossypium* and other eudicot species was demanded to explore how the BASS gene family has evolved in cotton. We, therefore, conducted the conserved synteny analysis which can help discover the origin of genes as neighborhood genes were likely persistent, even though target genes were lost during evolution^43^. *V. vinifera* not only has not undergone recent genome duplications but also is a palaeo-hexaploid common ancestor for many dicotyledonous plants including cotton^44^. *T. cacao* experienced fewer polyploidization events compared with cotton^45^, and it was very closely related to cotton (Fig. 2). For these reasons, *V. vinifera*, *T. cacao* and *Gossypium* spp. were used to trace the history of the BASS gene family in cotton. In a genome-wide comparison of BASS members among *V. vinifera*, *T. cacao* and *G. raimondii*, the same number of BASS1 should have been maintained in the genome of the last common ancestor of those species, meanwhile ancestral genes of BASS2, BASS3 and BASS5 were expanded two to three times only in *G. raimondii* after speciation from the last common ancestor (Fig. 4A,B). Interestingly, the ancestral gene of BASS4 was duplicated only in *V. vinifera* after speciation from the last common ancestor or perhaps the counterpart ortholog of this gene was lost in *T. cacao* and *G. raimondii* after speciation from the last common ancestor (There is a difficulty for this conclusion because BASS4 could not be mapped on syntenic blocks). Moreover, there was a case where BASS members were not mapped onto syntenic blocks, especially in *G. raimondii* vs. *V. vinifera* (Fig. 4A,B), suggesting that after speciation from the last common ancestor, each of those genomes evolved through the extensive genome fractionation accompanied by multiple rounds of chromosome breakages, rearrangements and fusions, and it was followed by the selective loss of genes, which makes a severe ambiguity in the identification of chromosomal syntenies^46^.

Regarding the synteny analysis between the *G. raimondii* and *G. arboretum* genome, ancestral genes of BASS1 and BASS2 were similarly extended to make two copies of those genes in both species after speciation from the last common ancestor, while ancestral genes of BASS3 and BASS5 were expanded two times in *G. arboretum*, but three times in *G. raimondii* after speciation from the last common ancestor (Fig. 4C). Contrary to other members, BASS4 was not found in the syntenic region, which is probably due to the imperfect genome assembly and annotation of *G. arboretum* because *GaBASS4* anchored in scaffold2621 (Fig. 4C). According to the synteny analysis between diploids and allotetraploids, some BASS family members showed the conservative evolution, e.g., BASS1 and BASS2 orthologs could maintain the conservative evolution, which was corroborated by the preserved collinearity of those orthologous gene pairs between diploids and allotetraploids (Fig. 4D,E). On the other hand, some members of BASS3 and BASS5 were not found in allotetraploids, even though they shared syntenic blocks (Fig. 4D,E). The A and D diploid ancestral species diverged from a common ancestor 5–10 million years ago^47^. And approximately 1.5 million years ago, these A- and D-genome progenitor were reunited by hybridization, as well as polyploidization events occurred^47^, which led to the evolution of the allotetraploid cotton. Thus, *G. hirsutum* and *G. barbadense* had undergone whole-genome duplications followed by the gene degeneration and loss, which causes a loss of some BASS family members in allotetraploids. For example, corresponding orthologs of *GrBASS3-2b*, *GrBASS5b* and *GrBASS5c* in the Dt-subgenome of *G. hirsutum*, as well as the orthologous counterpart gene of *GrBASS5c* in the Dt-subgenome of *G. barbadense* was lost. Unlikely, the conserved synteny of BASS4 was observed between the D-genome (*G. raimondii*) and Dt-subgenomes of allotetraploids, in spite of the fact that *GaBASS4* was not localized in the chromosome (Fig. 4D,E), which indicates that the *G. arboretum* genome database (BGI v1.0 assembly genome) has a flaw in relation to the BASS gene family, and if *GaBASS4* showed the shared synteny, it would really be helpful to trace the evolutionary history of BASS family members in cotton.

Studying the patterns of exon/intron organization can provide an additional insight into the structural architecture of genes^48^. In our results, not any big difference in patterns and structures of exon/intron was observed within a subfamily, but the distinct difference in their structures was found among subfamilies (Fig. 3B). Such a resembled gene structure in a subfamily suggests that *Gossypium* spp. underwent gene duplications and produced many copies of each subfamily member during their long evolutionary processes. Moreover, exon gains or exon losses occurred during their evolution. For instance, *GaBASS1-2* lost two exons, but *GaBASS5* gained one additional exon after speciation from the last common ancestor of *G. arboreum* and *G. raimondii*. With regard to the evolution from diploids to allotetraploids, BASSs of allotetraploids experienced both exon gains and losses (Fig. 3B). A motif is the most basic unit of a protein, and gene structure analyses based on motif patterns are increasingly demanded^25^. In our study, cotton BASSs with similar motif distribution patterns were closely clustered and made up a subfamily (Fig. 3A,C), hinting the high degree of sequence similarity within each subfamily, which reflects that BASS orthologs in each subfamily have similar functions and modes of action. Interestingly, each subfamily possessed unique motifs that probably have specific functions, that is, perhaps, the reason for a broad range of substrate specificity. Besides, Motif 1 and motif 2 on which two Na^+^-binding sites were situated (Fig. 3C) were distributed in almost all of the BASS members, indicating that cotton BASS genes are persistent to carry the two conserved Na^+^-binding sites.

### Functional characterization of *GhBASSs* in response to salt stress

Since the gene expression pattern is important information for the prediction of gene functions^49^, we characterized expression patterns of *GhBASSs* not only in different tissues but also under abiotic stresses and hormone elicitors. Except for *GhBASS4* which was ubiquitously expressed, other *GhBASS* genes exhibited the tissue-specific expression (Fig. 6). For example, *GhBASS1* and *GhBASS5* were highly expressed in roots (Fig. 6), hinting that these genes preferentially function in roots, i.e., functioning in the ion and mineral absorption and transportation from roots. By contrast, the transcripts of *GhBASS2* and *GhBASS3* were obviously high in leaf tissues (Fig. 6), which reflects that these genes participate in the functions of leaves. Abiotic stresses such as salt, drought and extreme temperatures seriously affect cotton growth and yield^15^, so our studies focused on molecular mechanisms concerning these stresses in cotton. In the present study, all *GhBASS* genes showed up- or down-regulation responding to different abiotic stresses (Fig. 7). This result can be speculated that different *GhBASS* genes play different functional roles in response to various abiotic stresses. ROS are important signal transduction molecules, as well as toxic oxidants which are by-products of stress metabolisms^50^. The ABA signaling involves in salinity and drought resistance of *Arabidopsis*^51^. In addition to having important roles in plant growth and development processes, GAs have regulatory functions in the plant cellular network of stress signaling^52^. SA is a phytohormone and improves the plant resistance to biotic and abiotic stresses^53^. Here, the elevated expression of *GhBASS* genes was imposed by various phytohormones, especially by ABA and MV (Fig. 8). This finding can be explained that *GhBASS* genes may involve in the signaling of many hormones, especially in ABA and ROS signal transduction pathways. Taken together, *GhBASS* genes might play a significant role in hormone signaling pathways which contribute to an interactive network that coordinates responses to different abiotic stresses. However, further studies are still needed to confirm the conclusions mentioned here.

Sodium is the main culprit of soil salinity, which is a major deleterious environmental factor limiting the global cotton production, and approximately 20% of the world’s agricultural land is presumably contaminated by Na^+^ ions^54^. The high level of soil salinity imposes osmotic stress and ion toxicity, which results in an imbalance in cellular homeostasis and functional disturbance of Ca^2+^ and K^+3^. Virus-induced gene silencing (VIGS) is a particularly useful tool for rapid and large-scale gene analyses in functional genomics and widely used to investigate the functions of genes^29^. BASS family members of different species retained the conserved Na^+^ binding sites^8,55^, which was consistent with our bioinformatics results (Figs. 3, S2). Besides, the transcriptional expression of *GhBASSs* (Fig. 7A) indicated that they tend to involve in salt stress. Hence, *GhBASS* genes were silenced and subjected to salt stress to uncover their functional roles concerning plant salt tolerance. In our results, *GhBASS1* and *GhBASS3* positively regulated, whereas *GhBASS2*, *GhBASS4* and *GhBASS5* negatively regulated the response of their corresponding knock-down plants to salt stress (Fig. 9). Na^+^ content is direct evidence for measuring the degree of salt stress in plants. Here, the silencing of *GhBASS1* and *GhBASS3* resulted in an increased Na^+^ accumulation, while the silencing of *GhBASS2*, *GhBASS4* and *GhBASS5* reduced Na^+^ content in comparison with the control (Fig. 10A), which matched up to the phenotypes of their corresponding knock-down plants (Fig. 9). In plants, Na^+^ is taken up at the root level, loaded into the xylem and then accumulated in the shoots, and Na^+^ transporters are key players in all of these steps^3^. BASS family genes are sodium-dependent metabolite co-transporters^7,8^. For example, *AtBASS2* was shown to mediate the co-transport of sodium and pyruvic acid into the chloroplast^9^, as well as *AtBASS5* was reported as a co-transporter of Na^+^ and 2-keto acids into plastids^11^. Next, *AtBASS1* localized in the plastid inner envelope and exported pantoate from the plastid^56^. However, the substrates of BASS3 and BASS4 are still unknown. Taken together, it is conceivable that *GhBASSs* are capable of transporting Na^+^, no matter what type of substrates they co-transport.

Since the subcellular localization of a protein is directly correlated to its function, the subcellular localization of GhBASS proteins will have important effects on their roles in relation to Na^+^ transportation^57^. To the best of our knowledge, all existing studies of plant BASS genes reported that they consistently located in chloroplast envelopes^9,10,12,56^. For instance, *Arabidopsis* BASS1 protein was solely localized in the plastid inner envelope^56^, *TaBASS2* located in the chloroplast membrane^13^ and *AtBASS5* specifically existed in the plastid envelope^10^. Consistently, GhBASS proteins were predominately localized in the chloroplast envelope (Fig. 5). Although the chloroplast contains its own genome, many of its genes are encoded in the nuclear genome. Most nuclear-encoded chloroplast proteins carry transit peptides (cTPs) at their N-terminus that guide them to the chloroplast^58^. Web server predictors such as ChloroP (A neural network-based method of predicting transit peptides)^58^ and LOCALIZER (A method for predicting plant and effector protein targeting to chloroplasts, mitochondria or nuclei)^59^ were developed for the prediction of a protein localization. By means of ChloroP (http://www.cbs.dtu.dk/services/ChloroP/) and LOCALIZER (http://localizer.csiro.au/), the chloroplast localization of GhBASSs were predicted because they held transit peptides (cTPs) (Fig. S5), which makes the experimentally subcellular localization result sound that GhBASSs are localized in chloroplast envelopes. Taken the sodium transportability and plastid-localized character of *GhBASSs* into consideration, it is speculated that *GhBASS2*, *GhBASS4* and *GhBASS5* import Na^+^ into chloroplasts, which is agreed by previous reports in which BASS2 and BASS5 genes co-transported Na^+^ and their respective substrates into chloroplasts^9–11,13^, while *GhBASS1* and *GhBASS3* export Na^+^ from chloroplasts, which is consistently found by Huang et al.^56^ that *AtBASS1* exported pantoate from the plastid by the Na^+^ dependent manner. This notion is further approved by the measurement of cytosolic and chloroplastic Na^+^ levels using the fluorescent dye SBFI-AM, as well as by the detection of net Na^+^ fluxes using Non-invasive Micro-test Technology (NMT) (Data not showed). Following this concept, when the expression of *GhBASS1* and *GhBASS3* is suppressed, their functions are weakened, probably resulting in more Na^+^ ions inside the chloroplast. By contrast, if *GhBASS2*, *GhBASS4* and *GhBASS5* are silenced, levels of Na^+^ ions inside the chloroplast will be feasibly decreased. Chloroplast serves as not only a factory for energy assimilation but also a generator for the synthesis of phytohormones, e.g., ABA and SA^13,60^ and important metabolites such as soluble sugar and proline^61^. In addition, chloroplast is one of the major sites for ROS production, and latter species of ROS can be produced in the plastid of root tissues also. The enhanced ROS generation in the chloroplast by salt stress changes the redox state of cells, which is instrumental in regulating the chloroplast metabolic activities^62,63^. Importantly, chloroplasts can be damaged by an excess of Na^+^ accumulated in chloroplasts^64^. Hence, chloroplast is a very important organelle in plant cells, and it is responsible for responding to salt stress^61^. Thereby, silencing of these genes affected the levels of Na^+^ accumulation inside chloroplasts, which in turn interfered with the functions of chloroplasts in their respective knock-down plants, and consequently, these knock-down plants suffered from salt stress with the varying degree of salt severity (Figs. 9, 10).

From our previous studies observed by Guo et al.^65^ and Myo et al.^66^, as well as literatures reported by Furumoto et al.^9^, Gigolashvili et al.^10^, Sawada et al.^11^ and Zhao et al.^13^, *GhBASS5* and *GhBASS2* are significantly promising candidate genes. Based on this information, we continued our study on these two genes employing the overexpression experiment. In our results, both of *GhBASS5* and *GhBASS2* impaired salt tolerance of transgenic *Arabidopsis* (Fig. 11), which was consistent with the increased Na^+^ accumulation in root tissues of OE plants (Fig. 12A). These results confirmed again that *GhBASS5* and *GhBASS2* are Na^+^ transporters, as well as negatively regulate the plant salt tolerance, which is agreed with the gene silencing results (See the above paragraphs). With regard to the sodium-coupled substrate transported by BASS5, Sawada et al.^11^ presented that *AtBASS5* co-transported Na^+^:2-keto acids into the chloroplast for the synthesis of Glucosinolates (GSLs). Moreover, Hossain et al.^67^ stated that GLS degradation products stimulated the generation of ROS which can cause oxidative stress if they are at the excessively accumulated level. Taken *GhBASS5’s* co-transporter role into consideration, if the *GhBASS5* gene is overexpressed, high levels of Na^+^ and 2-keto acids will be expectedly accumulated inside the chloroplast, which will harm the functions of the chloroplast. By this means, disturbing chloroplast functions resulted in an impaired tolerance of the plant to salt stress (Figs. 11, 12). If *GhBASS5* serves as a co-transporter of Na^+^ and 2-keto acids, a question of what biosynthesis pathway occurs in cotton will be interested in this context because GSL compounds are restricted to the Brassicaceae^11^. Pyruvic acid is the precursor of abscisic acid (ABA), and ABA is generated inside the chloroplast^13,61^. ABA acts as an endogenous messenger functioning in the regulating and signaling network of salt response^68^. Regarding the BASS2’s symporter model of Na^+^:pyruvic acids, Furumoto et al.^9^ reported that *AtBASS2* imported pyruvate into the plastid via the Na^+^ dependent manner. From the view of the sodium-coupled pyruvate transporter role of *GhBASS2*, it co-transports a couple of pyruvic acids and Na^+^ ions into the chloroplast. Accordingly, pyruvic acids and Na^+^ ions should be highly accumulated inside the chloroplasts of *GhBASS2*-OE plants because the numbers of *GhBASS2*’s transcripts are multiplied in those overexpressing experiments. As a result, such an excess of Na^+^ accumulated inside the chloroplast damages the functions of the chloroplast, rendering a weakened tolerance of the plant against salt stress (Figs. 11, 12). However, there is a cross-talk that *TaBASS2* served as a pyruvate transporter into the chloroplast, which resulted in salt tolerance of wheat^13^. In such a case, more ABA will be possibly synthesized by the chloroplast, and there will be similarly more Na^+^ ions inside the chloroplast, but they will be under the detrimental level. Logically, the accumulated levels of pyruvate and Na^+^ inside the chloroplast should have the threshold level. In accordance with this notion, if they are above this level, the excessive accumulation of Na^+^ will disturb the functions of chloroplasts, even though ABA can enhance the plant tolerance to salt stress. Accordingly, it is comprehensible that *GhBASS2* might play a dual role, and whether having the role of positive or negative regulation in plant salt tolerance may depend on the expression levels of this gene. Although this report cannot explore exactly where Na^+^ was accumulated, i.e., in the cytoplasm or plastid, how a plastidial sodium-dependent transporter regulated other genes participated in the pathway network responding to salt stress and what detailed metabolic mechanisms occurred concerning salt stress for each gene, this paper is the second report in relation to a plastid-localized sodium-coupled metabolite transporter that can influence Na^+^ transportation and plant salt tolerance, which is fundamental information for further detailed analyses of salt-tolerant mechanisms.

In conclusion, BASS family genes are very conserved across all tested organisms, and *GhBASSs* are sodium-dependent metabolite co-transporters. To the best of our knowledge, this paper is the second report to validate that a plastidial Na^+^-coupled metabolite transporter carries out Na^+^ transporting function in plants and has a powerful impact on plant salt tolerance. We believe that our findings will provide programs for the production of salt-tolerant cotton varieties with fundamental information, even though precise metabolic mechanisms of *GhBASS* genes concerning salt stress require further investigation.

## Materials and methods

All the experiments have been done in accordance with relevant institutional, national, and international guidelines and legislation.

### Database search and identification of BASS family genes from cotton and other species

Genes and proteins annotated in *G. arboretum* (BGI v1.0 assembly genome), *G. raimondii* (JGI v2.1 assembly genome) and *G. hirsutum* (NAU-NBI v1.1 assembly genome) were downloaded from the CottonGen database (www.cottongen.org), but genomic sequences of *G. barbadense* were downloaded from http://database.chgc.sh.cn/cotton/index.html. Genomic sequences of *Arabidopsis thaliana* were downloaded from the TAIR database (https://www.arabidopsis.org/), *Oryza stiva* were from the MSU Rice Genome Annotation Project database (http://rice.plantbiology.msu.edu/), *Picea abies* from the TreeGenes database (https://treegenesdb.org/org/Picea-abies), *Phalaenopsis equestris* from the PLAZA database (https://bioinformatics.psb.ugent.be/plaza/versions/plaza_v4_monocots/) and *Nicotiana tabacum* from the Sol Genomics Network database (https://solgenomics.net/organism/ Nicotiana_tabacum/genome). While *Rhizophora apiculata*, *Neisseria meningitidis*, *Escherichia coli*, *Yersinia frederiksenii*, *Micromonas pusilla*, *Homo sapiens*, *Mus musculus*, *Oryctolagus cuniculus* and *Rattus rattus* sequences were retrieved from the NCBI RefSeq database (http://www.ncbi.nlm.nih.gov/), genomic sequences of other species were retrieved from the Phytozome 12.1 (https://phytozome.jgi.doe.gov/pz/portal.html). BASS family genes were identified from various genomes by using the HMMER software version 3.0 and the file on the Hidden Markov Model profile of the Pfam SBF domain (PF01758) (http://pfam.xfam.org) as a query. For gene loci having many isoforms, the primary isoform was selected if the primary isoform annotation was available; otherwise, the longest one was chosen. After removing partial and short sequences, the deduced sequences were examined whether they harbored a bass and an SBF domain by NCBI Conserved Domain Database (NCBI-CDD) (https://www.ncbi.nlm.nih.gov/Structure/cdd/wrpsb.cgi) and Pfam (http://pfam.xfam.org/), followed by the observation of transmembrane regions and Na^+^-binding sites using protocols described by Zhou et al.^8^. All predicted BASSs were named based on the method described by Mohanta et al.^69^, where BASSs from monocot plant species were named in accordance with the BASS orthologs of *Oryza sativa*, whereas BASSs from other species were named according to BASS orthologs of *Arabidopsis thaliana*.

### Multiple sequence alignment, phylogenetic analysis and gene architectures

Multiple sequence alignment of BASS proteins from different organisms was conducted employing the online software MAFFT through the EMBL-EBI bioinformatics interface with default parameters^70^. Poorly aligned positions and divergent regions resulting from multiple sequence alignment were eliminated by Gblocks to get conserved blocks (http://molevol.cmima.csic.es/castresana/Gblocks_server.html)^71^ using the following parameters: (i) smaller final blocks, (ii) gap positions within the final blocks and (iii) less strict flanking positions. The phylogenetic tree was built by the PhyML (http://phylogeny.lirmm.fr/), where Approximate Likelihood-Ratio Test (aLRT) was chosen as statistical tests for the branch support, and LG (aa) was used as a substitution model^72^. The output tree in Newick format was visualized with the iTOL v4 (http://itol.embl.de/)^70^. The local version of Multiple Em for Motif Elicitation (MEME) v4.12.0 was applied with default parameters for the identification of conserved motifs across cotton proteins^74^. Information for distribution of exons, introns and coding sequences was obtained from gff3 files of cotton genome annotation data. Finally, gene architectures were depicted using the TBtools-JRE1.6 software^75^.

### Synteny and Ka/Ks analysis

Synteny blocks containing BASS genes between different genomes and/or subgenomes of cotton species, as well as between cotton and other plant species were searched employing the MCScanX software^76^. Each BASS gene was correctly mapped onto the corresponding chromosomes of *Gossypium* spp., *Vitis vinifera* and *Theobroma cacao* according to the retrieved information from gff3 files of genome annotation data. The blocks and collinearity of homologous gene pairs were depicted by performing the CIRCOS program^77^. On the basis of codon level alignment of coding sequences, nonsynonymous to synonymous substitution ratios (Ka/Ks) of all orthologous, paralogous and homoeologous gene pairs were calculated by the software KaKs_Calculator Version 2.0 with the method of MA and model averaging^78,79^.

### Plant materials, growth conditions and treatments

Seeds of salt-sensitive upland cotton cv. Zhong G5, which were kindly provided by the Institute of Cotton Research of CAAS (Anyang, China), were delinted and germinated in the plastic tray having wet sand. Cotton seedlings with two cotyledons were transplanted to hydroponic pots containing 1/2 Hoagland nutrient solution, then maintained them under the controlled conditions (25 °C with 16/8 h light/dark). For the tissue-specific expression analysis, leaves, stems and roots of four-leaf-stage cotton seedlings were harvested for RNA extraction. Three-week-old cotton seedlings were used for the treatments of abiotic stresses and hormone elicitors. For signaling substance treatments, leaves were sprayed with 0.3 mM methyl viologen (MV), 2 mM salicylic acid (SA), 1 mM abscisic acid (ABA) and 0.5 mM gibberellic acid (GA3). Roots of cotton seedlings were irrigated with 200 mM NaCl for the salt treatment and 10% PEG 6000 (W/V) for the drought stress. For temperature stress treatments, seedlings were kept inside a growth chamber at a high temperature (37 °C) or a low temperature (4 °C). For all treatments, control plants were applied with ddH_2_O. Regarding the plant materials in this experiment, 16 plants for one gene for one replication for each treatment were applied to a completely randomized design (CRD) with two replications. Accordingly, the experiment was repeated three successive times. Samples were collected at 0, 0.2, 3, 6, 12, 24, 48, 72 and 96 h post-treatment (hpt). After harvesting, samples were immediately frozen in liquid nitrogen and stored at − 80 °C until RNA extraction.

*Arabidopsis thaliana* ecotype Columbia (Col-0) was employed to play as a wild-type (WT) plant. Seeds of loss-of-function mutant of *AtBASS5* (SALK_126525) and loss-of-function mutant of *AtBASS2* (SALK_101808) were obtained from the Nottingham Arabidopsis Stock Centre (NASC)/Arabidopsis Biological Resource Center (ABRC). SALK_126525 mutant, as well as SALK_101808 mutant was induced by pROK2 T-DNA insertion^80^. Plant materials for transformation were grown in pots with well-watered vermiculite and humus mix inside a greenhouse (22 °C, 100 μmol photons m^−2^ s^−1^, 60% relative humidity and 16/8 h day/night cycles).

### RNA extraction, cDNA synthesis and qPCR analyses

Total RNA was extracted using the Plant Total RNA Isolation Kit Plus (Foregene, Chengdu, China) with the manufacturer’s guidelines. The TransScript One-Step gDNA Removal and cDNA Synthesis SuperMix kit (TransGen Biotech, Beijing, China) was used for the first-strand cDNA synthesis following the supplier’s protocols. The relative quantification of *GhBASSs* by qPCR was conducted with SYBR Green on a LightCycler 480 (Roche Molecular Systems, Inc., Indianapolis, Indiana, USA). Following the instructions of the TransStart Tip Green qPCR SuperMix kit (TransGen Biotech, Beijing, China), the reaction mixture and PCR cycles were performed. The cotton UBQ 7 (GenBank accession no. DQ116441) and *Arabidopsis* UBQ 10 (GenBank accession no. AT4G05320) were applied as internal references for normalization. The transcript levels of *GhBASSs* were calculated by the comparative 2^−ΔΔCT^ method^81^, and three independent biological replicates were done for each assay. Primers used for qPCR analyses were listed in Table S3.

### Cloning and subcellular localization of GhBASSs-GFP fusion proteins

All *GhBASS* genes were amplified from cotton with specific primers (Table S3) designed from the identified deduced sequences resulting from bioinformatics analyses. The PCR amplified products were independently cloned into the pMD19-T Vector (Takara Biomedical Technology Co., Ltd., Beijing, China) following the manufacturer’s instructions. The cloned full-length cDNA sequences of *GhBASSs* were examined by performing the alignment analysis with their corresponding query sequences derived from bioinformatics studies. Next, *GhBASS* genes were separately inserted into the pCaMV35S:GFP vector with the use of specific primers (Table S3) to generate pCaMV35S:GhBASSs:GFP recombinant plasmids. Then, not only those fusion proteins (pCaMV35S:GhBASSs:GFP) but also the empty vector control (pCaMV35S:GFP) were transiently expressed in *A. thaliana* mesophyll protoplasts using the PEG-mediated transformation method^27^. After incubating the PEG transfected mesophyll protoplasts in the dark for 16 h, GFP fluorescence was observed under a confocal laser scanning microscope (Carl Zeiss Co., Ltd., Jena, Germany).

### Agrobacterium-mediated virus-induced gene silencing and salt tolerance analysis

The construction of tobacco rattle virus (TRV)-based VIGS vectors and inoculation of *Agrobacterium tumefaciens* strain GV3101 harboring recombinant TRV vectors were performed as described previously^30^. Briefly, cDNA fragments of *GhCLA1* (420 bp) and *GhBASSs* (250–450 bp) were amplified by PCR with specific primers (Table S3). The resulting PCR products were separately cloned into pTRV2 to produce recombinant vectors, pTRV:*GhCLA1* and pTRV:*GhBASSs*. These recombinant vectors were independently introduced into *A. tumefaciens* strain GV3101, and then cultured at 28 °C for 24 h. Afterword, *Agrobacterium* carrying pTRV1 was independently mixed with *Agrobacterium* having recombinant vector pTRV:*GhCLA1* or pTRV:*GhBASSs* in a 1:1 ratio just before inoculated into cotyledons of cotton seedlings. TRV:GFP was included as a negative control, whereas TRV:*GhCLA1* served as a positive control. The agroinfiltrated plants were maintained in a greenhouse (23 °C, 120 μmol photons m^-2^ s^-1^ light, 12 h light/12 h dark cycle). After the photobleaching phenotype of TRV:*GhCLA1* had been observed, gene silencing efficiency was verified by examining the expression level of endogenous BASS genes using RT-PCR and qPCR. The silenced plants were then subjected to 200 mM NaCl treatment for the salt-tolerant test. Regarding the plant materials in VIGS experiments, 16 plants for one gene for one replication were applied to a completely randomized design (CRD) with two replications. Accordingly, the results were validated by three successive times.

### Transgenic plant generation and salt tolerance analysis

To construct pCAMBIA1302:*GhBASS2* and pCAMBIA1302:*GhBASS5* recombinant vectors, each of *GhBASS2* and *GhBASS5* was separately introduced into the pCAMBIA1302 vector under the control of a cauliflower mosaic virus 35S promoter. *Agrobacterium tumefaciens* strain GV3101 harboring pCAMBIA1302:*GhBASS2* or pCAMBIA1302:*GhBASS5* was separately transformed into *A. thaliana* (Col-0) by the floral dip method with minor modifications^82^. Transgenic T1 seedlings were screened by growing on the 1/2 MS medium supplemented with 50 μg ml^−1^ hygromycin, and surviving seedlings on the selective medium were validated by PCR. To confirm whether these selected T1 lines were independent with different T-DNA insertion loci in the *Arabidopsis* genome, they were then screened by thermal asymmetric interlaced PCR (TAIL-PCR) following previously described methods^36–38^ with some modifications. Of these independent transgenic lines, two T3 homozygous lines obtained from T2 lines showing the correct segregation ratio (3:1) were confirmed by qPCR, as well as RT-PCR and then the correct ones were selected for further functional studies. Seedlings and adults of WT, loss-of-function mutants and T3 homozygous transgenic lines were exposed to salt stress for the observation of salt-stressed symptoms and salt-tolerant phenomenon. Primers used in this study were included in Table S3. Regarding the plant materials in these experiments, 30 seedlings/plants for one overexpressing line for one replication were applied to a completely randomized design (CRD) with two replications. Accordingly, the results were validated by three successive times.

### ***Measurement of Na***^+^***and K***^+^***contents***

Salt-treated and none salt-treated plants were sampled for the measurement of their ions concentration as described by Rus et al.^83^ with some modifications. In brief, harvested samples were dried inside a drying oven at 70 °C for 4 days. The oven-dried samples were digested with 0.1 M HNO_3_ and boiled at 90 °C for an hour, then filtered through the Whatman no. 1 filter paper. Na^+^ and K^+^ content in the filtrated solutions were determined by an atomic absorption spectrophotometer (Triad Scientific, Inc., Manasquan, New Jersey, USA).

### Statistical analysis

To perform statistical analysis, IBM SPSS version 16.0 (SPSS Inc., Armonk, New York, USA) and GraphPad Prism version 8.0 (GraphPad Software Inc., San Diego, CA, USA) were used. Data come from three independent biological replicates were analyzed by applying independent sample t-test or one-way ANOVA based on Duncan’s multiple range test. The significant difference level was signified with a single star (*, *P* = 0.05).

## Data availability statement

The data in relation to this study are obtainable from corresponding authors on reasonable request.

## Permission statement for plant materials

All required approvals for collection of plant or seed specimens were obtained for the study, which complied with relevant institutional, national, and international guidelines and legislation.

## Supplementary Information

Supplementary Information 1.

Supplementary Information 2.

Supplementary Information 3.

Supplementary Information 4.
