# Supplementary material for: Genome-wide identification of the BASS gene family in four Gossypium species and functional characterization of GhBASSs against salt stress
Source: Sci Rep. 2021 May 31;11:11342. doi: 10.1038/s41598-021-90740-3 (PMC8166867; doi:10.1038/s41598-021-90740-3)
Supplement: Supplementary file 1 — Supplementary Information 1. [file 41598_2021_90740_MOESM1_ESM.pdf]

Supplementary materials

Gossypium arboreum

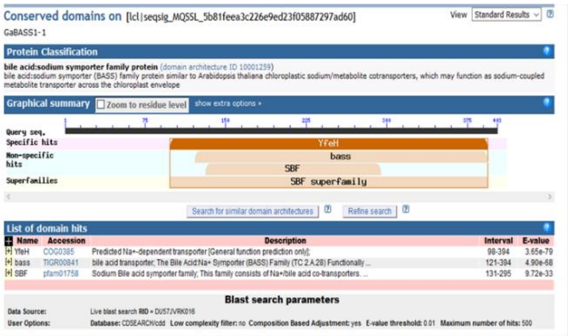

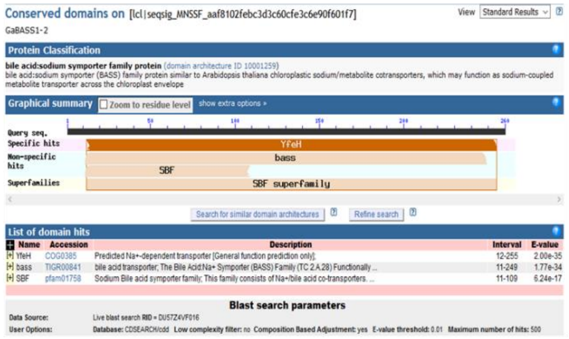

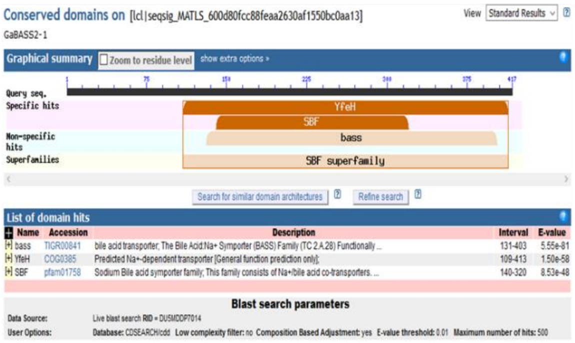

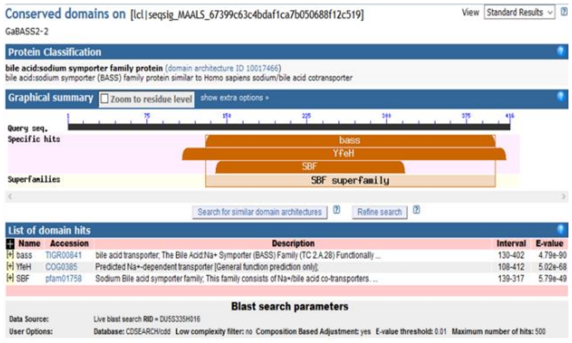

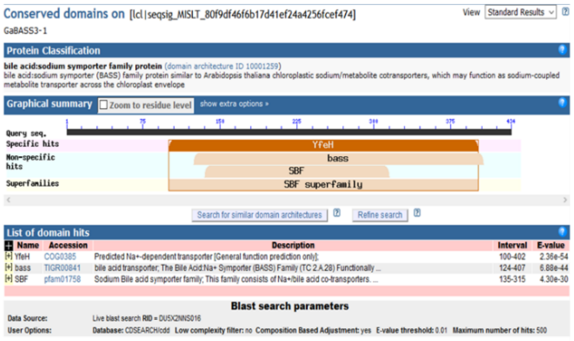

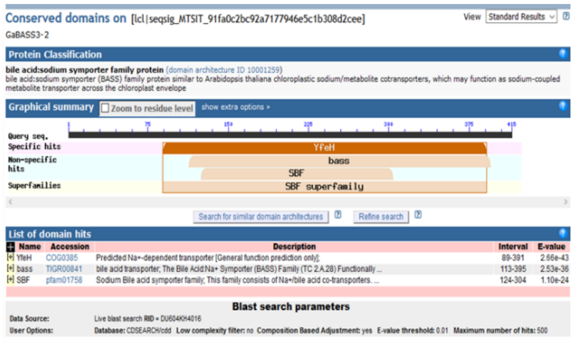

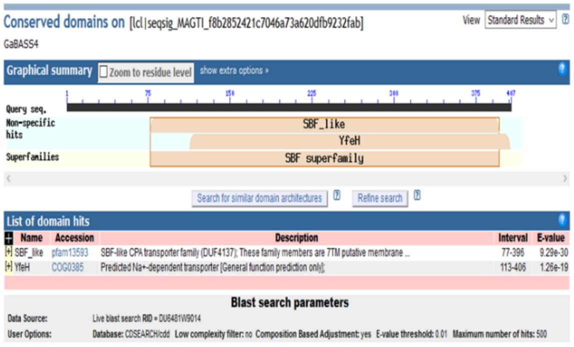

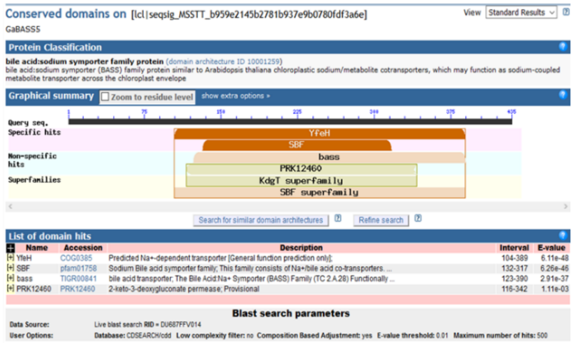

Gossypium raimondii

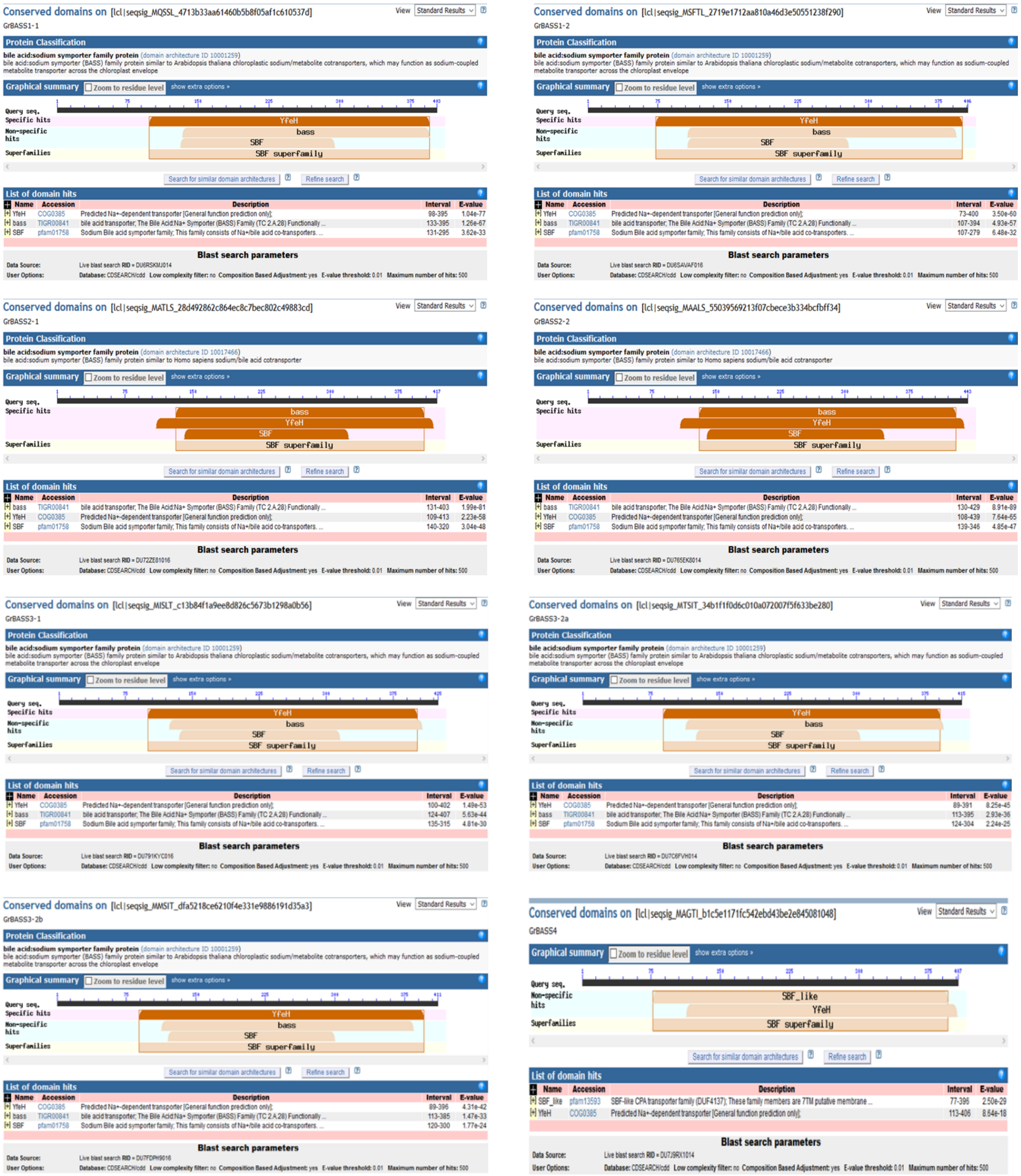

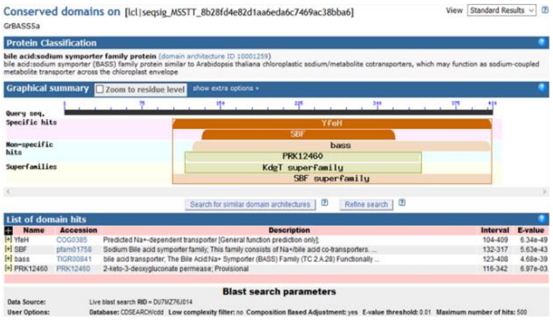

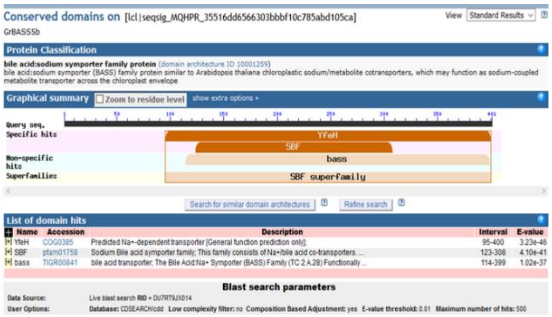

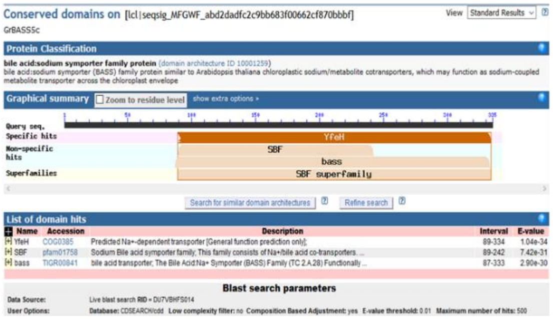

Gossypium hirsutum

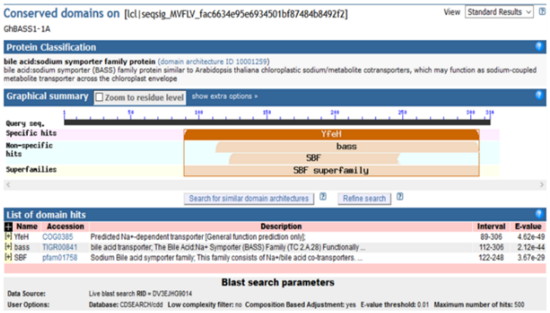

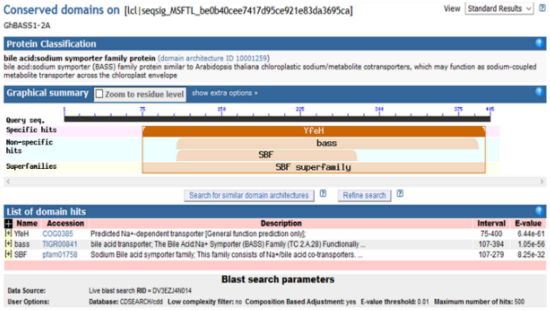

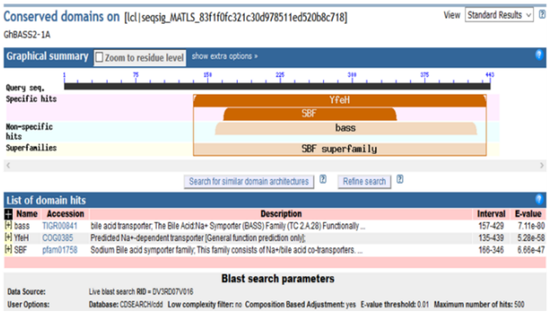

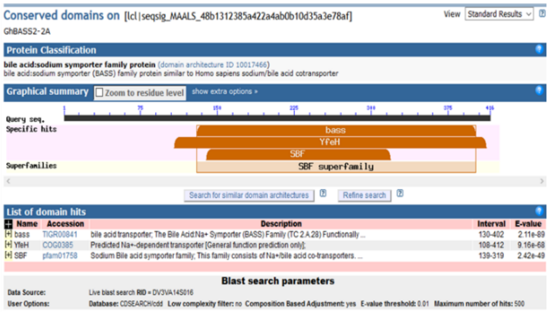

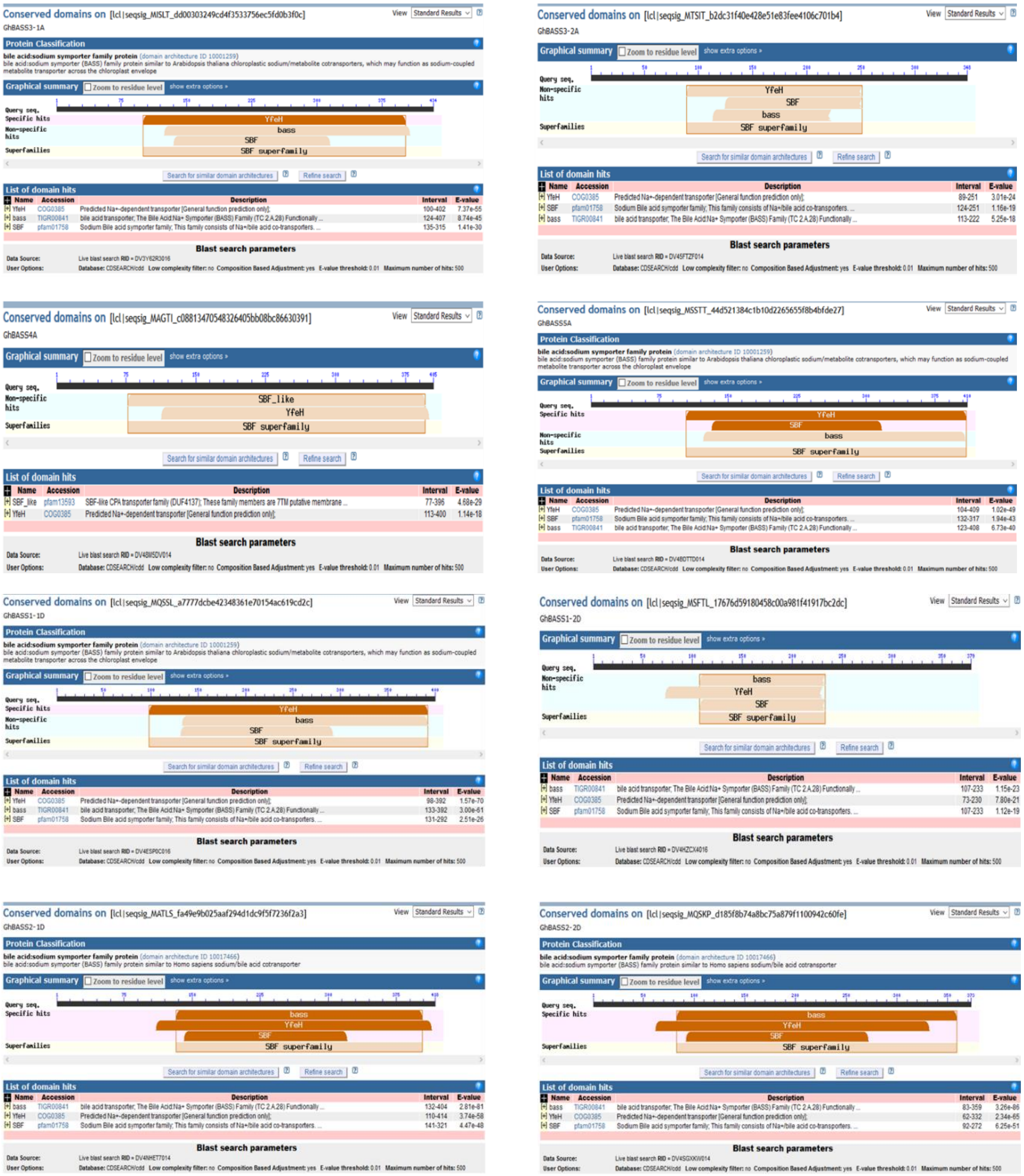
Conserved domains on [lcl|seqsig_MISLT_dd00303249cd4f3533756ec5fd0b3f0c]
GhBASS3-1A
Conserved domains on [lcl|seqsig_MTSIT_b2dc31f40e428e51e83fee4106c701b4]
GhBASS3-2A
Conserved domains on [lcl|seqsig_MAGTI_c08813470548326405bb08bc86630391]
GhBASS4A
Conserved domains on [lcl|seqsig_MSSTT_44d521384c1b10d2265655f8b4bfde27]
GhBASS5A
Conserved domains on [lcl|seqsig_MQSSL_a7777dcbe42348361e70154ac619cd2c]
GhBASS1-1D
Conserved domains on [lcl|seqsig_MSFTL_17676d59180458c00a981f41917bc2dc]
GhBASS1-2D
Conserved domains on [lcl|seqsig_MATLS_fa49e9b025aaf294d1dc9f5f7236f2a3]
GhBASS2-1D
Conserved domains on [lcl|seqsig_MQSKP_d185f8b74a8bc75a879f1100942c60fe]
GhBASS2-2D
Protein Classification
Graphical summary
List of domain hits
Blast search parameters
YfeH
bass
SBF
SBF superfamily
SBF_like

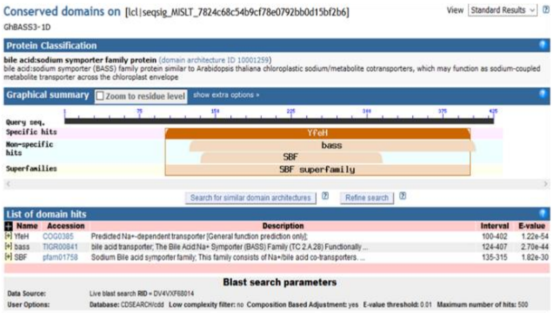

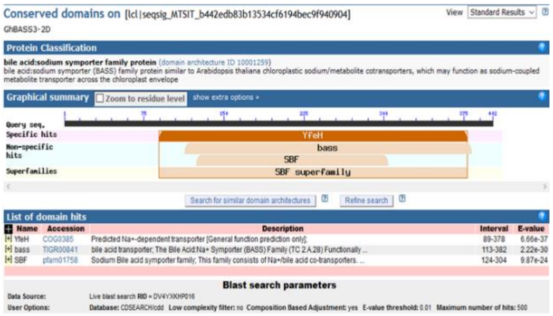

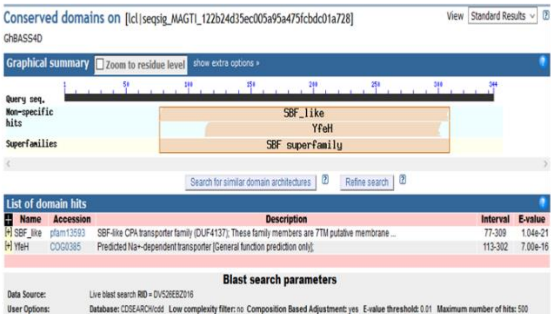

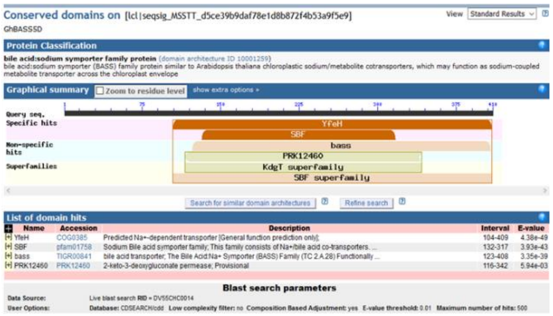

Gossypium barbadense

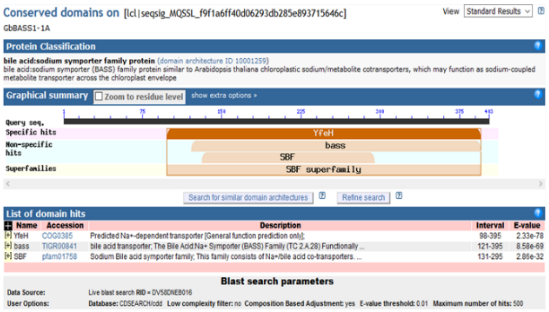

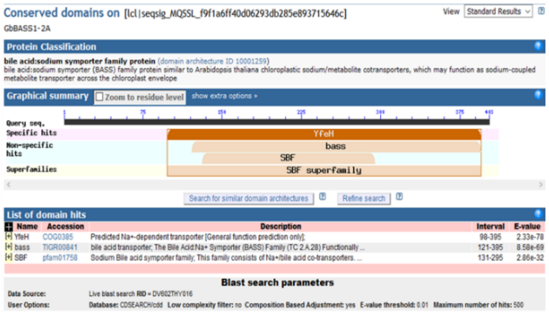

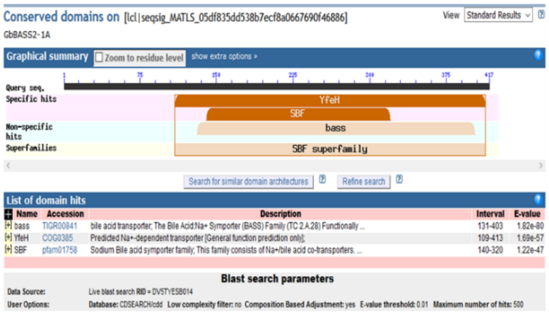

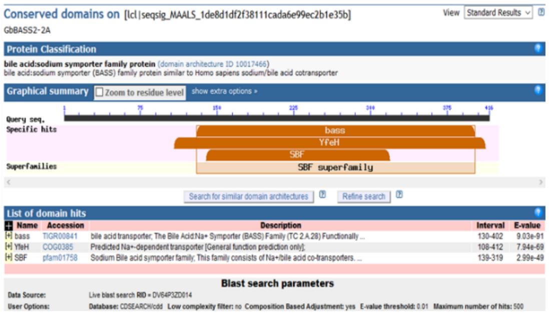

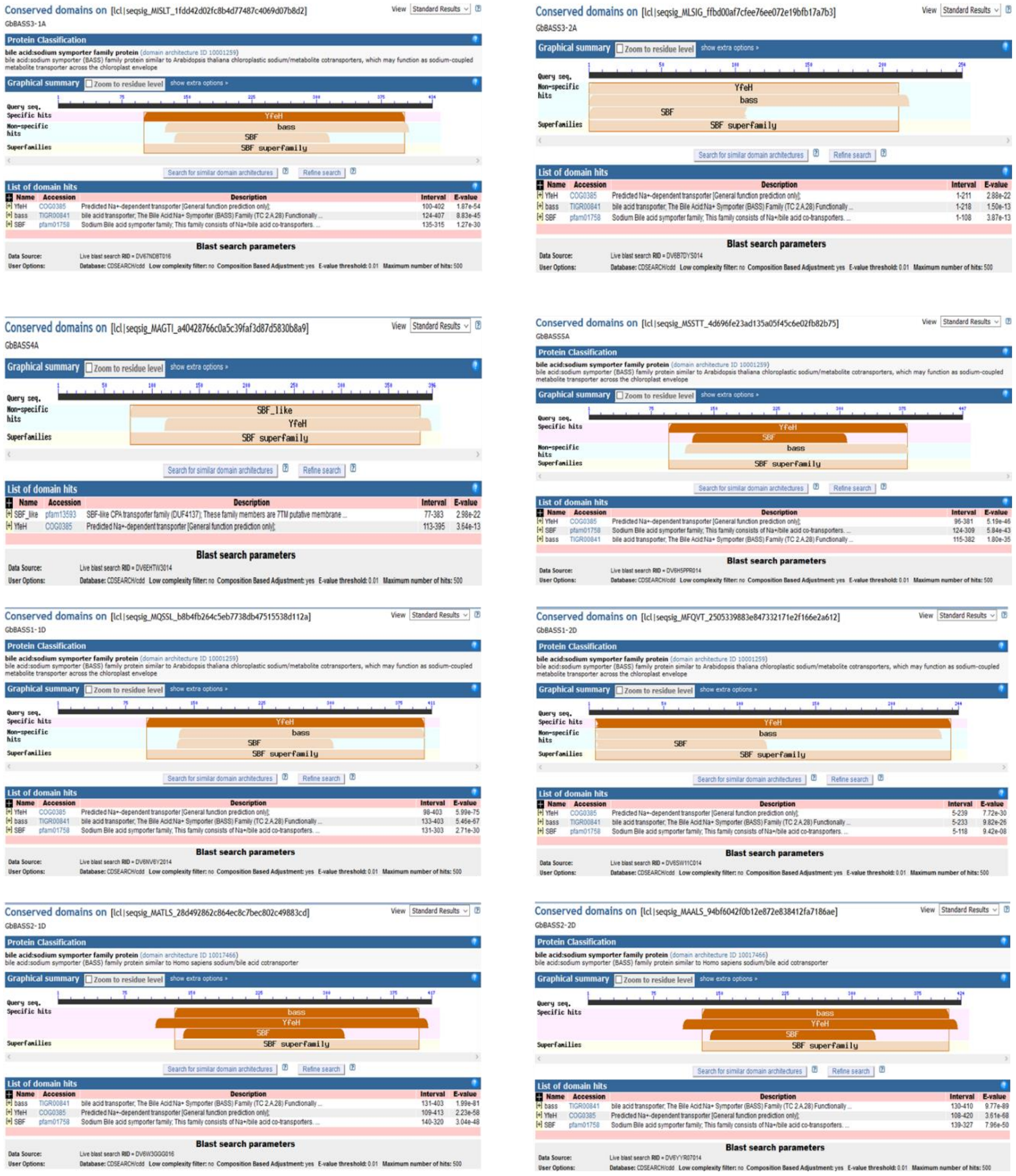
Conserved domains on [lcl|seqsig_MISLT_1fdd42d02fc8b4d77487c4069d07b8d2]
GbBASS3-1A
Protein Classification
bile acid:sodium symporter family protein (domain architecture ID 10001259)
bile acid:sodium symporter (BASS) family protein similar to Arabidopsis thaliana chloroplastic sodium/metabolite cotransporters, which may function as sodium-coupled metabolite transporter across the chloroplast envelope
Graphical summary
Query seq.
Specific hits
Non-specific hits
Superfamilies
YfeH
bass
SBF
SBF superfamily
List of domain hits
Name Accession Description Interval E-value
YfeH COG0385 Predicted Na+-dependent transporter [General function prediction only]; 100-402 1.87e-54
bass TIGR00841 bile acid transporter; The Bile Acid:Na+ Symporter (BASS) Family (TC 2.A.28) Functionally ... 124-407 8.83e-45
SBF pfam01758 Sodium Bile acid symporter family; This family consists of Na+/bile acid co-transporters. ... 135-315 1.27e-30
Blast search parameters
Data Source: Live blast search RID = DV67NDBT016
User Options: Database: CDSEARCH/cdd Low complexity filter: no Composition Based Adjustment: yes E-value threshold: 0.01 Maximum number of hits: 500
Conserved domains on [lcl|seqsig_MLSIG_ffbd00af7cfee76ee072e19bfb17a7b3]
GbBASS3-2A
YfeH COG0385 Predicted Na+-dependent transporter [General function prediction only]; 1-211 2.88e-22
bass TIGR00841 bile acid transporter; The Bile Acid:Na+ Symporter (BASS) Family (TC 2.A.28) Functionally ... 1-218 1.50e-13
SBF pfam01758 Sodium Bile acid symporter family; This family consists of Na+/bile acid co-transporters. ... 1-108 3.87e-13
Data Source: Live blast search RID = DV6B7DYS014
Conserved domains on [lcl|seqsig_MAGTI_a40428766c0a5c39faf3d87d5830b8a9]
GbBASS4A
SBF_like
SBF_like pfam13593 SBF-like CPA transporter family (DUF4137); These family members are 7TM putative membrane ... 77-383 2.98e-22
YfeH COG0385 Predicted Na+-dependent transporter [General function prediction only]; 113-395 3.64e-13
Data Source: Live blast search RID = DV6EHTW3014
Conserved domains on [lcl|seqsig_MSSTT_4d696fe23ad135a05f45c6e02fb82b75]
GbBASS5A
YfeH COG0385 Predicted Na+-dependent transporter [General function prediction only]; 96-381 5.19e-46
SBF pfam01758 Sodium Bile acid symporter family; This family consists of Na+/bile acid co-transporters. ... 124-309 5.84e-43
bass TIGR00841 bile acid transporter; The Bile Acid:Na+ Symporter (BASS) Family (TC 2.A.28) Functionally ... 115-382 1.80e-35
Data Source: Live blast search RID = DV6H5PPR014
Conserved domains on [lcl|seqsig_MQSSL_b8b4fb264c5eb7738db47515538d112a]
GbBASS1-1D
YfeH COG0385 Predicted Na+-dependent transporter [General function prediction only]; 98-403 5.99e-75
bass TIGR00841 bile acid transporter; The Bile Acid:Na+ Symporter (BASS) Family (TC 2.A.28) Functionally ... 133-403 5.46e-67
SBF pfam01758 Sodium Bile acid symporter family; This family consists of Na+/bile acid co-transporters. ... 131-303 2.71e-30
Data Source: Live blast search RID = DV6NV6Y2014
Conserved domains on [lcl|seqsig_MFQVT_2505339883e847332171e2f166e2a612]
GbBASS1-2D
YfeH COG0385 Predicted Na+-dependent transporter [General function prediction only]; 5-239 7.72e-30
bass TIGR00841 bile acid transporter; The Bile Acid:Na+ Symporter (BASS) Family (TC 2.A.28) Functionally ... 5-233 9.82e-26
SBF pfam01758 Sodium Bile acid symporter family; This family consists of Na+/bile acid co-transporters. ... 5-118 9.42e-08
Data Source: Live blast search RID = DV6SW11C014
Conserved domains on [lcl|seqsig_MATLS_28d492862c864ec8c7bec802c49883cd]
GbBASS2-1D
bile acid:sodium symporter family protein (domain architecture ID 10017466)
bile acid:sodium symporter (BASS) family protein similar to Homo sapiens sodium/bile acid cotransporter
bass TIGR00841 bile acid transporter; The Bile Acid:Na+ Symporter (BASS) Family (TC 2.A.28) Functionally ... 131-403 1.99e-81
YfeH COG0385 Predicted Na+-dependent transporter [General function prediction only]; 109-413 2.23e-58
SBF pfam01758 Sodium Bile acid symporter family; This family consists of Na+/bile acid co-transporters. ... 140-320 3.04e-48
Data Source: Live blast search RID = DV6W3GGG016
Conserved domains on [lcl|seqsig_MAALS_94bf6042f0b12e872e838412fa7186ae]
GbBASS2-2D
bass TIGR00841 bile acid transporter; The Bile Acid:Na+ Symporter (BASS) Family (TC 2.A.28) Functionally ... 130-410 9.77e-89
YfeH COG0385 Predicted Na+-dependent transporter [General function prediction only]; 108-420 3.61e-68
SBF pfam01758 Sodium Bile acid symporter family; This family consists of Na+/bile acid co-transporters. ... 139-327 7.96e-50
Data Source: Live blast search RID = DV6YYR07014

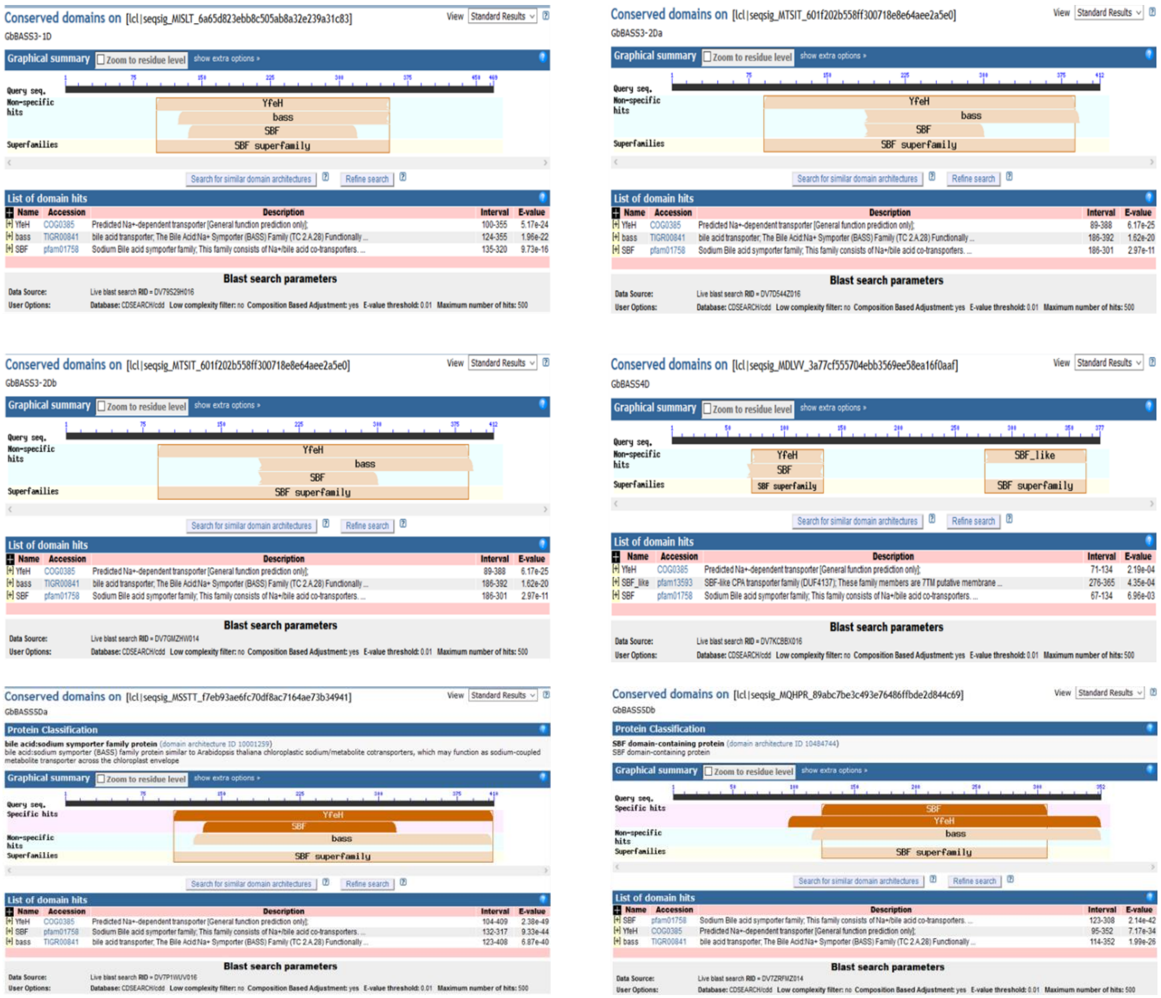

Figure S1. The conserved SBF and bass domain of cotton BASS gene family members. The deduced sequences identified from *Gossypium* spp. were examined whether they harbored a bass and an SBF domain by NCBI-CDD (https://www.ncbi.nlm.nih.gov/Structure/cdd/wrpsb.cgi) and Pfam (http://pfam.xfam.org/). BASS, bile acid sodium symporter; *Ga*, *Gossypium arboreum*; *Gr*, *Gossypium raimondii*; *Gb*, *Gossypium barbadense*; *Gh*, *Gossypium hirsutum*.

Gossypium arboreum

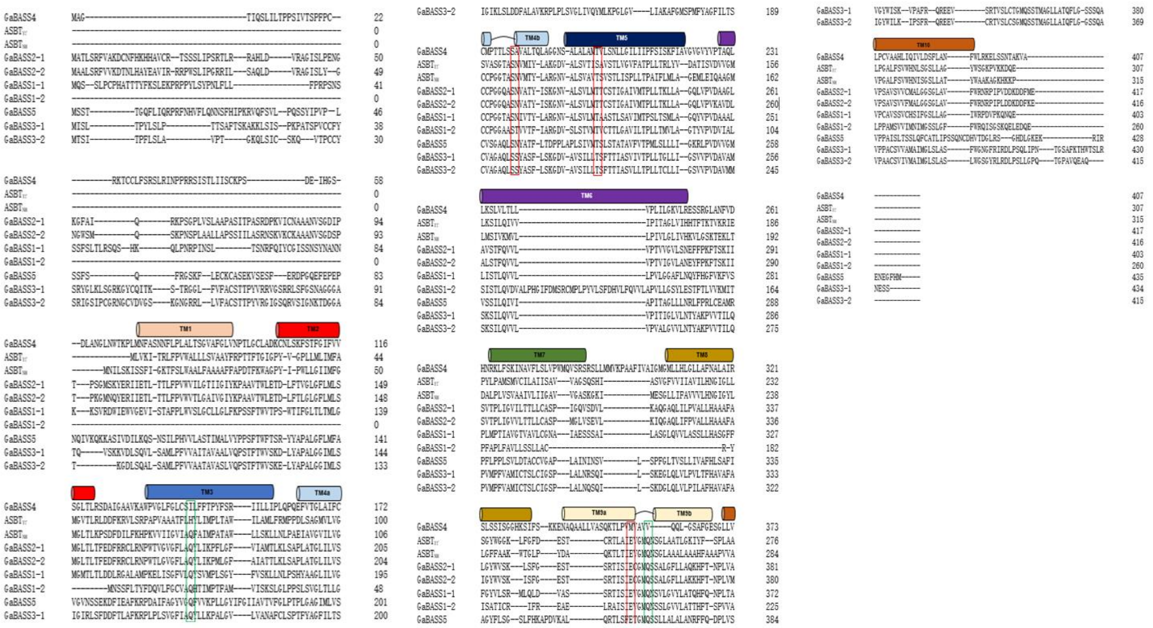

Gossypium raimondii

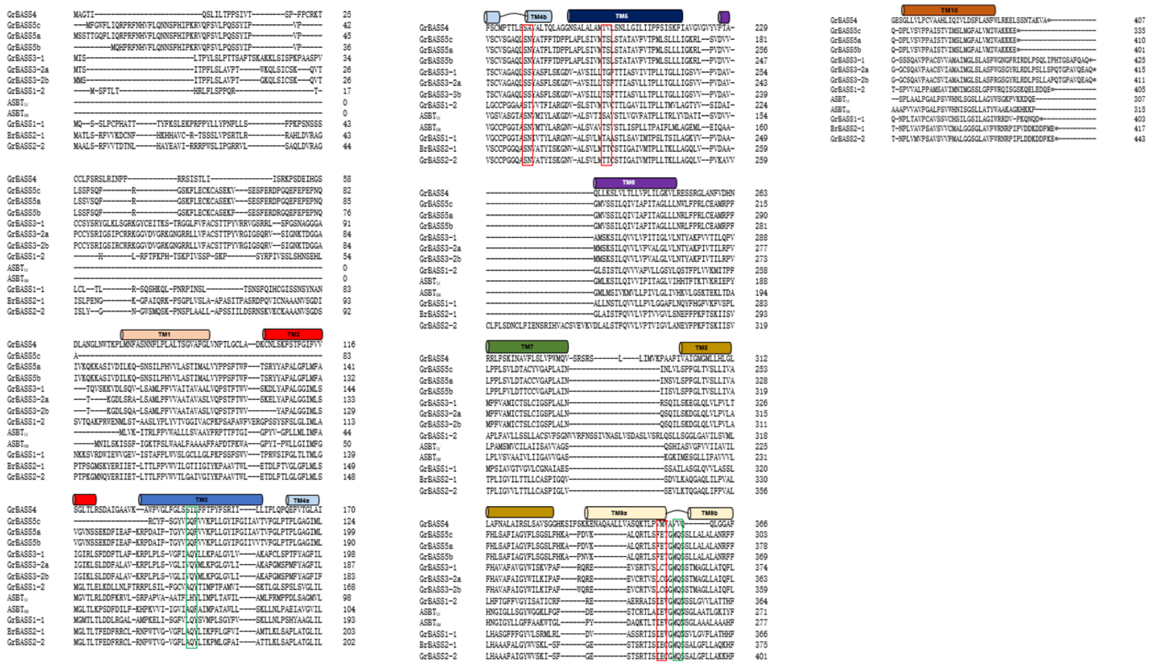

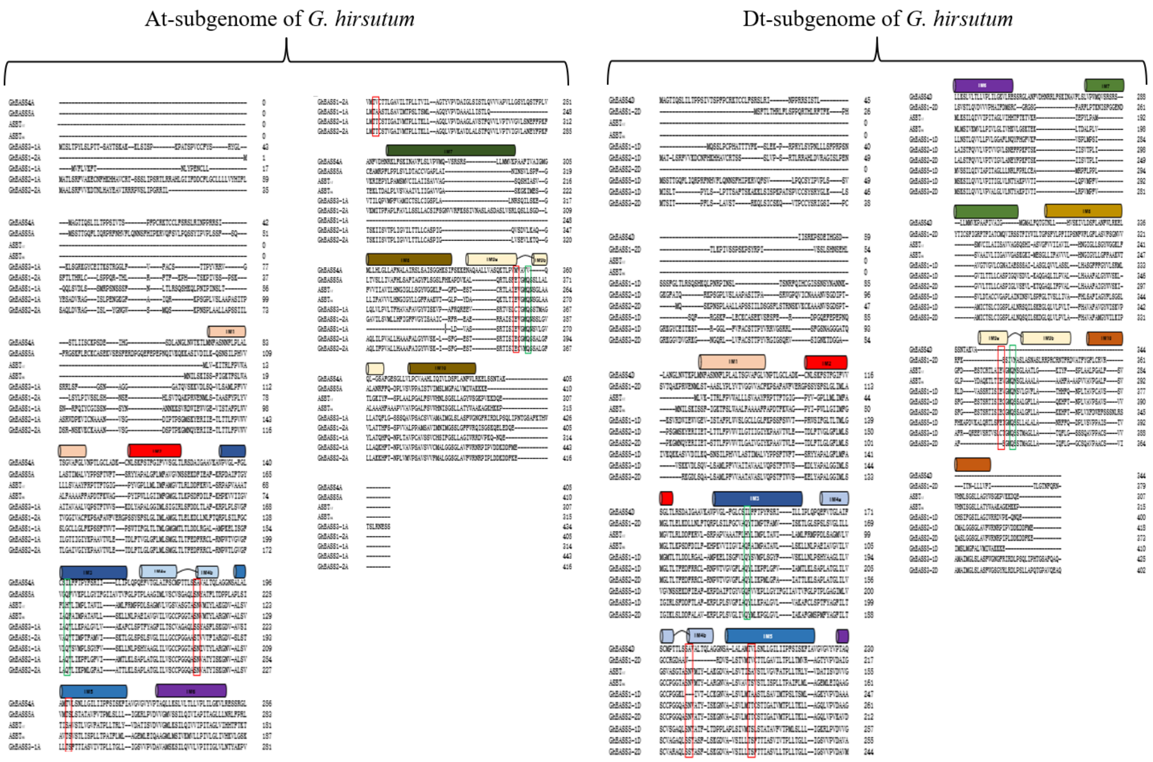
At-subgenome of *G. hirsutum*
Dt-subgenome of *G. hirsutum*

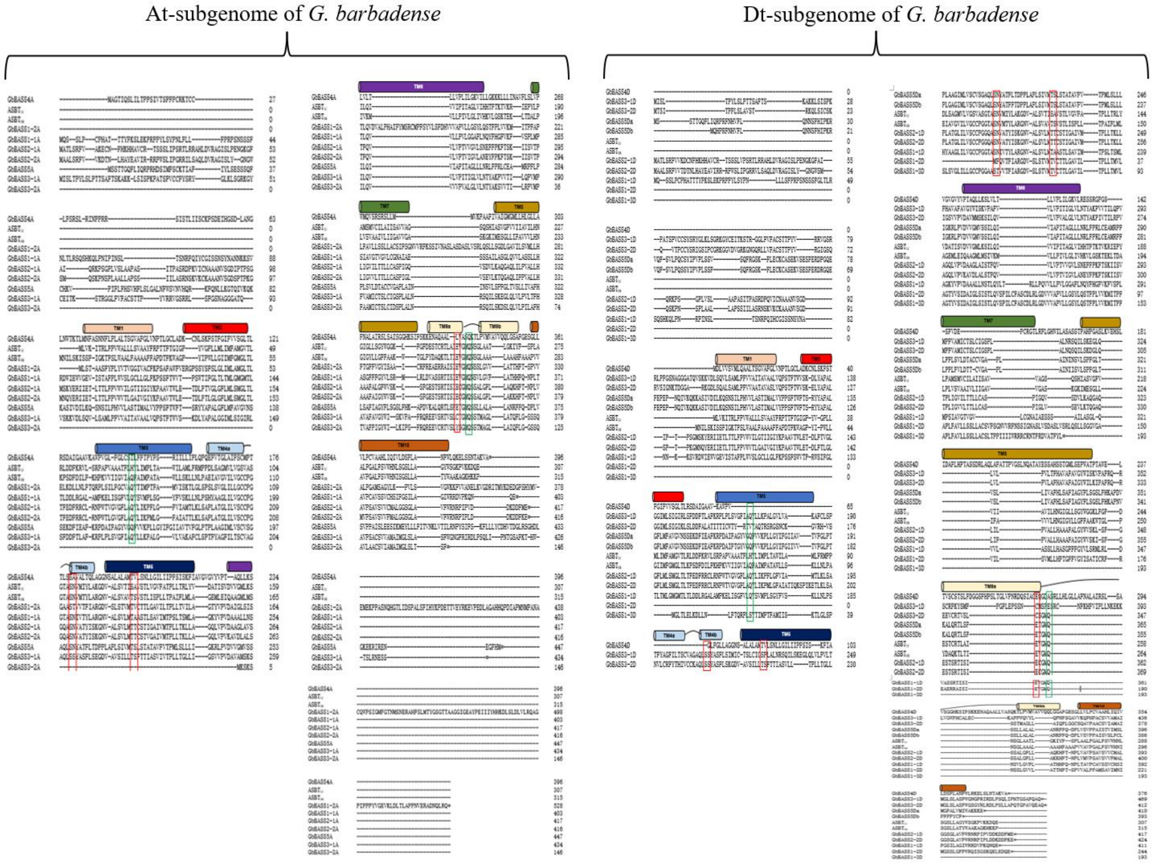

Figure S2. Sequence conservation of cotton BASS gene family members. Multiple sequence alignment was performed by the online MAFFT program and DNAMAN 6.0.3.99. The colored bars represent the locations of transmembrane helices, and Na1 and Na2 binding sites are marked with the red and green rectangular boxes, respectively. *Ga*, *Gossypium arboreum*; *Gr*, *Gossypium raimondii*; *Gb*, *Gossypium barbadense*; *Gh*, *Gossypium hirsutum*.

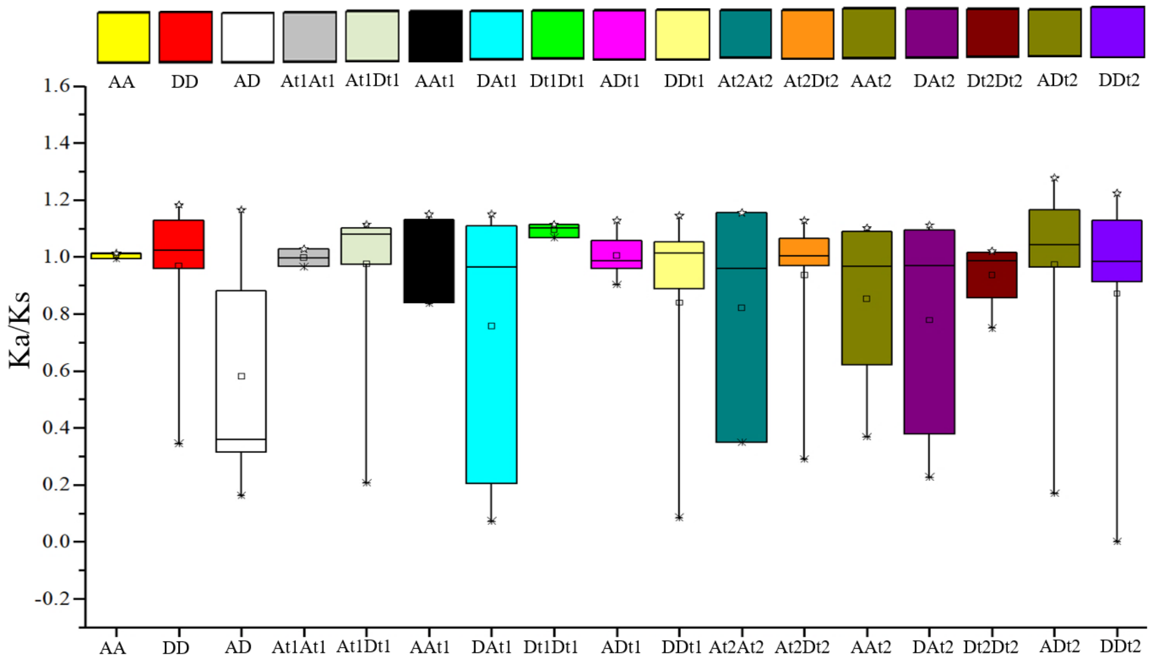

Figure S3. The Ka/Ks values of the homologous/homoeologous BASS gene pairs among four cotton species. The Ka/Ks values of the homologous/homoeologous BASS genes pairs among the genomes and/or subgenomes of *G. arboretum* (A), *G. raimondii* (D), *G. hirsutum* (At1Dt1) and *G. barbadense* (At2Dt2). The bars show variation among data, the symbol (✮) presents the maximum value, the symbol (✳) explains the minimum value, the symbol (□) represents the mean value and the line across the box is median.

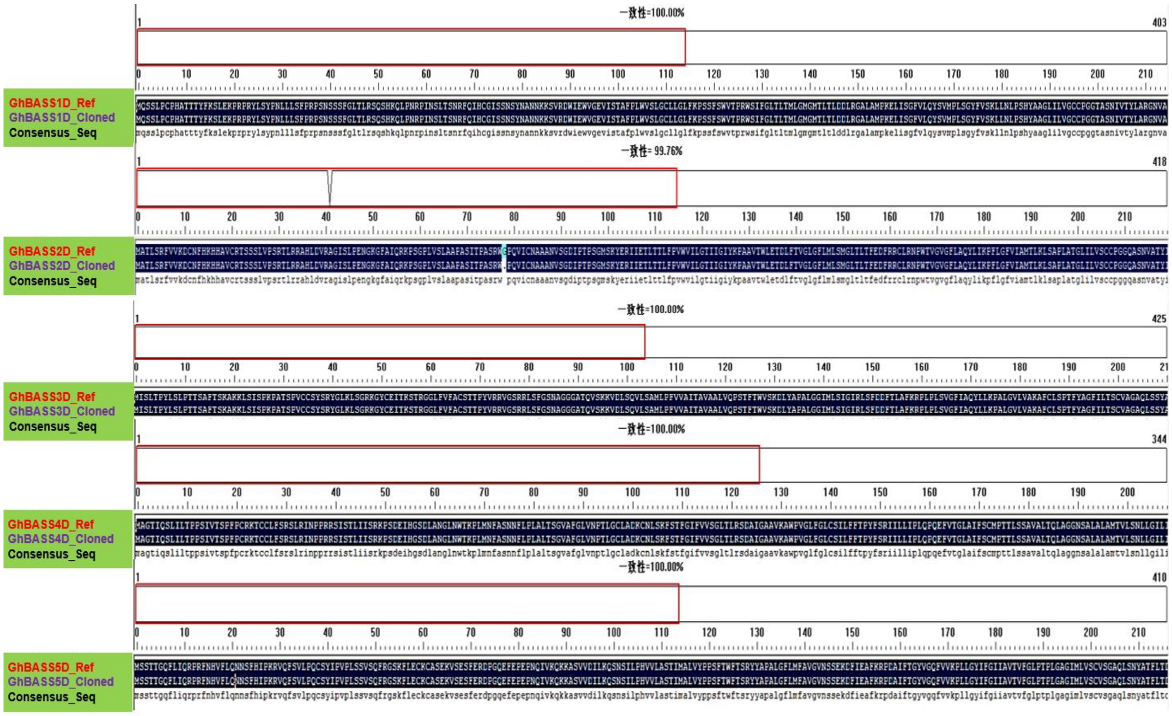

Figure S4. Protein sequence analysis of cloned *GhBASS* genes. Sequencing was performed using DNAMAN 6.0.3.99 with the default parameters.

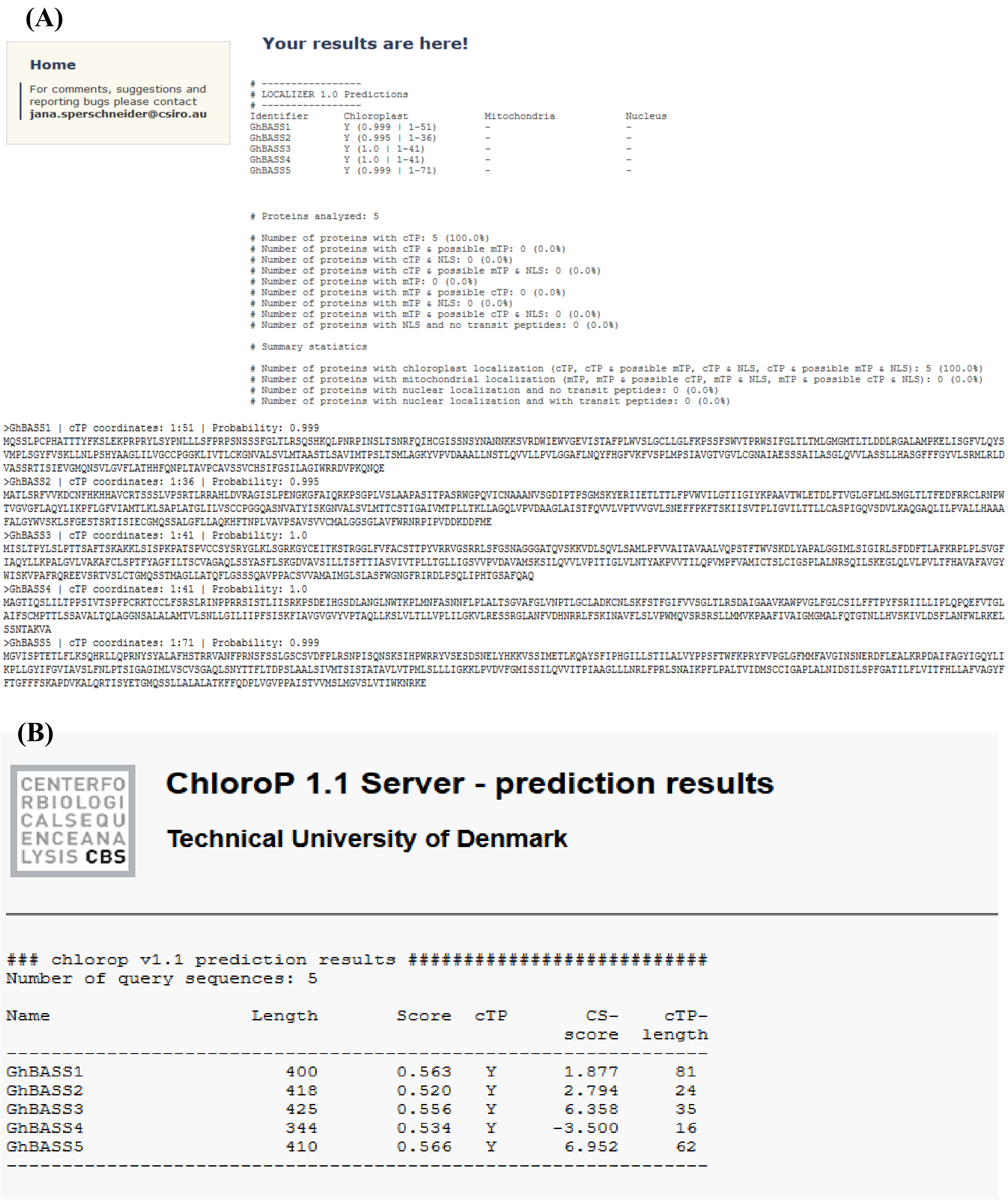

Figure S5. The chloroplast localization of GhBASSs predicted by LOCALIZER and ChloroP. (**a**) The chloroplast localization of GhBASSs was predicted by LOCALIZER which predicts chloroplast and mitochondrial transit peptides or nucleus localization signals in plant proteins. (**b**) The chloroplast localization of GhBASSs was predicted by ChloroP which predicts chloroplast transit peptides. Y, the sequence is predicted to contain a cTP; cTP, chloroplast transit peptide; mTP, mitochondrial transit peptide; NLS, nucleus localization signal.

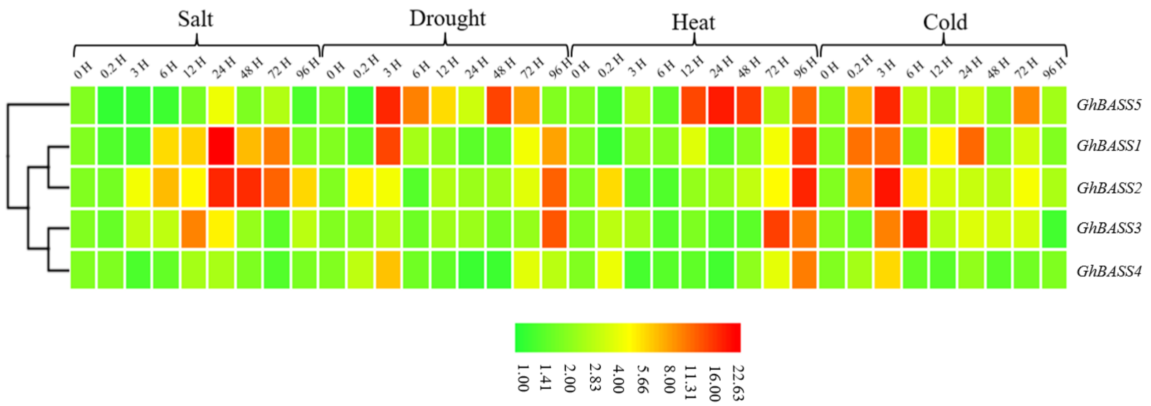

Figure S6. Heatmap of *GhBASSs* expression under different abiotic stresses. Expression patterns by heatmap were depicted using the TBtools-JRE1.6 software.

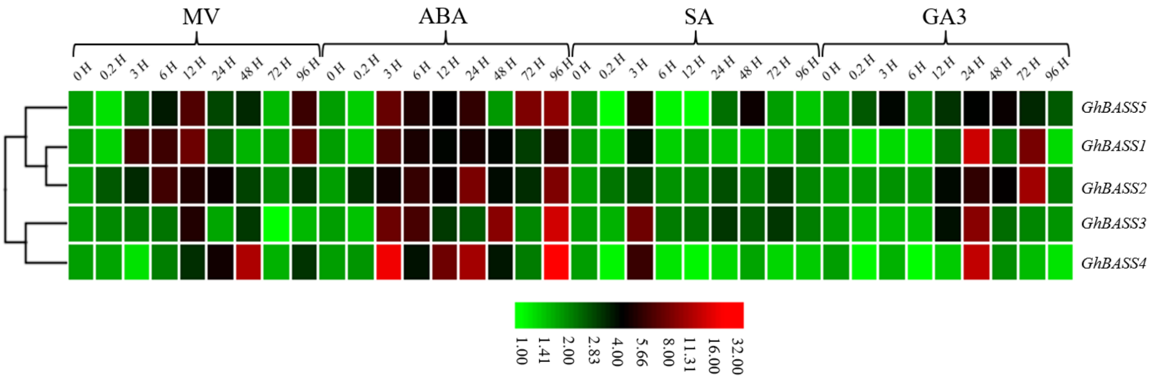

Figure S7. Heatmap of *GhBASSs* expression under the treatments of various phytohormones. Expression patterns by heatmap were depicted using the TBtools-JRE1.6 software. MV, methyl viologen; ABA, abscisic acid; SA, salicylic acid; GA3, gibberellic acid.

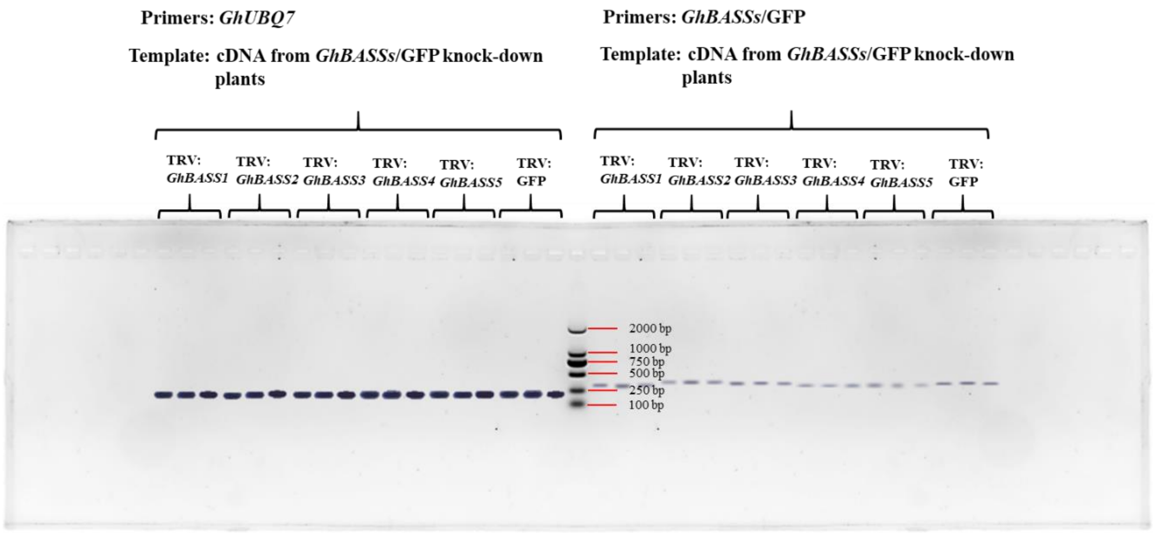

Figure S8. Related transcript levels of *GhBASSs* in *GhBASSs* knock-down plants by RT-PCR. Gene silencing efficiency was verified by examining the expression level of endogenous BASS genes by RT-PCR using gene-specific primers (Table S3) and cDNA template from *GhBASSs* knock-down plants. *GhUBQ7* (GenBank accession no. DQ116441) was used as a control.

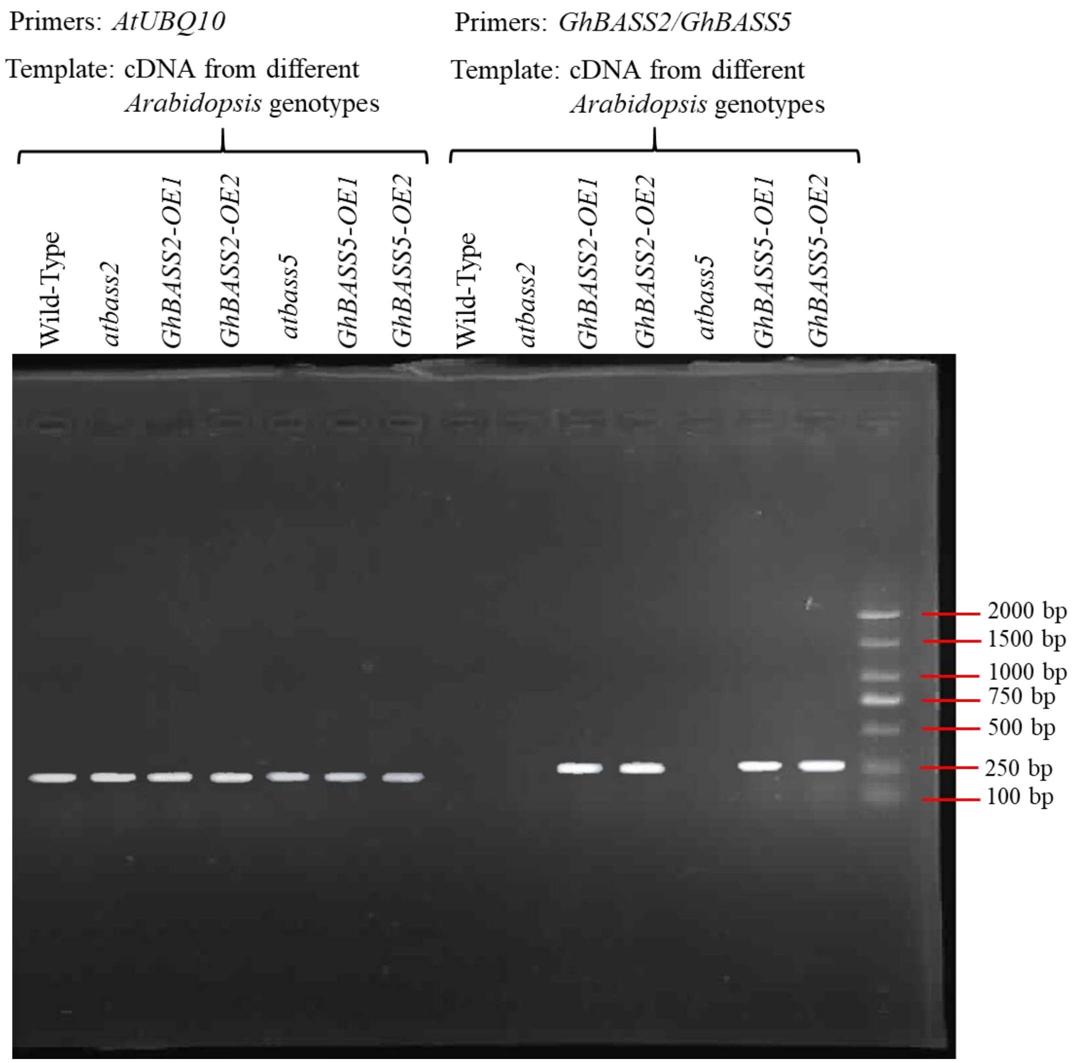

Figure S9. Transcript levels of *GhBASS2* and *GhBASS5* in wild-type, mutants and OE plants by RT-PCR. T3 homozygous lines obtained from T2 lines showing the correct segregation ratio (3:1) were confirmed by RT-PCR using gene-specific primers (Table S3) and cDNA template from different *Arabidopsis* genotypes. The *AtUBQ10* gene (GenBank accession no. AT4G05320) was used as a control.

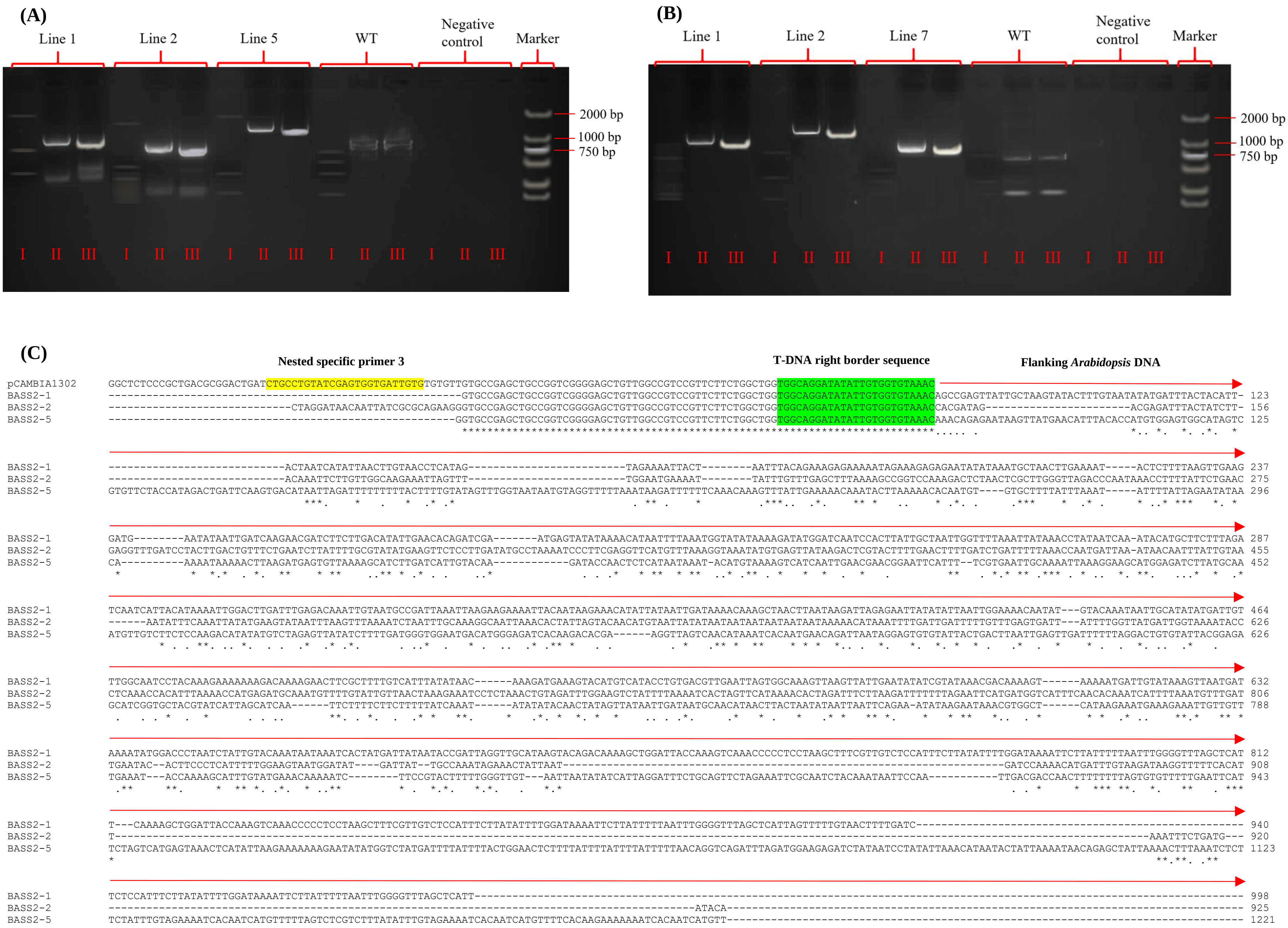
(A)
Line 1
Line 2
Line 5
WT
Negative control
Marker
2000 bp
1000 bp
750 bp
I II III I II III I II III I II III I II III
(B)
Line 1
Line 2
Line 7
WT
Negative control
Marker
2000 bp
1000 bp
750 bp
I II III I II III I II III I II III I II III
(C)
Nested specific primer 3
T-DNA right border sequence
Flanking Arabidopsis DNA
pCAMBIA1302
BASS2-1
BASS2-2
BASS2-5

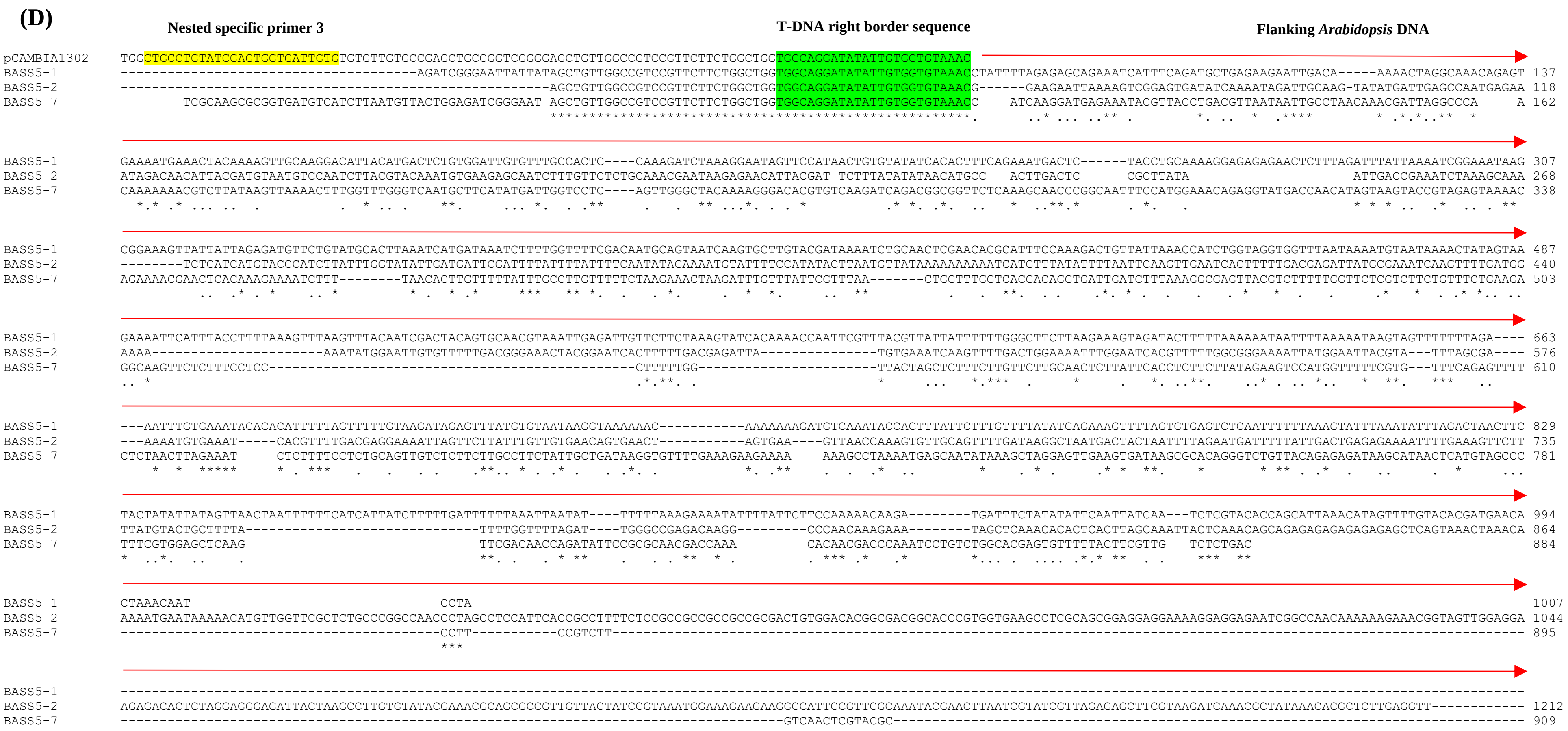

Figure S10. Amplification and sequencing the sequences flanking the T-DNA insertion site by thermal asymmetric interlaced PCR (TAIL-PCR). (**A**) Agarose gel electrophoresis of TAIL-PCR products for three *GhBASS2*-OE transgenic lines. (**B**) Agarose gel electrophoresis of TAIL-PCR products for three *GhBASS5*-OE transgenic lines. I, primary TAIL-PCR products; II, secondary TAIL-PCR products; III, tertiary TAIL-PCR products. (**C**) Sequence alignment of three transgenic lines of *GhBASS2*-OE plants together with the right T-border region of the pCAMBIA1302 vector positioned on the first line. (**D**) Sequence alignment of three transgenic lines of *GhBASS5*-OE plants together with the right T-border region of the pCAMBIA1302 vector positioned on the first line. Sequences of transgenic lines homologous to the T-DNA sequence of the right border side from pCAMBIA1302 vector are marked with the green colour. The yellow-coloured highlight is the nested specific primer 3 (SP3) which was exploited as the sequencing primer. Red arrow indicates the sequences flanking the T-DNA insertion site isolated from three *GhBASS2*-OE or *GhBASS5*-OE transgenic lines. The symbols "* and ." indicate sequence similarity across the transgenic lines.
